# Toxicity and safety study of silver and gold nanoparticles functionalized with cysteine and glutathione

**DOI:** 10.3762/bjnano.10.175

**Published:** 2019-09-02

**Authors:** Barbara Pem, Igor M Pongrac, Lea Ulm, Ivan Pavičić, Valerije Vrček, Darija Domazet Jurašin, Marija Ljubojević, Adela Krivohlavek, Ivana Vinković Vrček

**Affiliations:** 1 Institute for Medical Research and Occupational Health, Ksaverska cesta 2, 10000 Zagreb, Croatia; 2 University of Zagreb, School of Medicine, Croatian Institute for Brain Research, Šalata 12, 10000 Zagreb, Croatia; 3 Andrija Štampar Teaching Institute of Public Health, Mirogojska 16, 10000 Zagreb, Croatia; 4 Faculty of Pharmacy and Biochemistry, University of Zagreb, Ante Kovačića 1, 10000 Zagreb, Croatia; 5 Rudjer Boskovic Institute, Bijenička cesta 54, 10000 Zagreb, Croatia

**Keywords:** biocompatibility, cysteine, ecotoxicity, glutathione, nano–bio interactions, nanosafety, nanotoxicity

## Abstract

This study was designed to evaluate the nano–bio interactions between endogenous biothiols (cysteine and glutathione) with biomedically relevant, metallic nanoparticles (silver nanoparticles (AgNPs) and gold nanoparticles (AuNPs)), in order to assess the biocompatibility and fate of nanoparticles in biological systems. A systematic and comprehensive analysis revealed that the preparation of AgNPs and AuNPs in the presence of biothiols leads to nanoparticles stabilized with oxidized forms of biothiols. Their safety was tested by evaluation of cell viability, reactive oxygen species (ROS) production, apoptosis induction and DNA damage in murine fibroblast cells (L929), while ecotoxicity was tested using the aquatic model organism *Daphnia magna*. The toxicity of these nanoparticles was considerably lower compared to their ionic metal forms (i.e., Ag^+^ and Au^3+^). The comparison with data published on polymer-coated nanoparticles evidenced that surface modification with biothiols made them safer for the biological environment. In vitro evaluation on human cells demonstrated that the toxicity of AgNPs and AuNPs prepared in the presence of cysteine was similar to the polymer-based nanoparticles with the same core material, while the use of glutathione for nanoparticle stabilization was considerably less toxic. These results represent a significant contribution to understanding the role of biothiols on the fate and behavior of metal-based nanomaterials.

## Introduction

Metallic nanoparticles (NPs) such as silver and gold have been employed in a wide range of products and applications in the biomedical field owing to their remarkable physico-chemical properties. Silver nanoparticles (AgNPs) are extensively used in antimicrobial coatings for medical devices, wound dressing, cosmetic products and food packaging due to their antimicrobial, antiangiogenic and anti-inflammatory properties [1–5]. Biomedical applications of gold nanoparticles (AuNPs) range from molecular imaging, targeted drug delivery, gene therapy, cancer treatment or radio-sensitization and theranostics [1,4,6]. Moreover, AgNPs and AuNPs are among the most investigated engineered nanomaterials for medical use. A search performed in the Web of Science (WoS) database on November 11th 2018 using the search terms “nano*” and “medic*” yielded a total of 63,032 papers. Out of those, silver was reported in 6,004 papers (9.5%) and gold in 8,757 (13.9%), revealing that these two types of NPs alone are represented by almost one quarter of all published studies on biomedical aspects of NPs (Figure S1 in Supporting Information File 1). In addition to the increase of papers reporting on the development of NPs for biomedical uses, the WoS search showed that there are ample in vitro and in vivo studies on the toxicity effects of AgNPs and AuNPs. There are a number of studies indicating that AgNPs negatively impact cell membranes, interfere with signaling pathways, disrupt the cell cycle, and cause mitochondrial dysfunction, oxidative stress, DNA damage and apoptosis [7–9]. Many reports on AgNP toxicity attribute it fully or partially to dissolved or released ionic silver [10–16]. The dissolution of AgNPs in both biological and in different environmental media has been proven, although the rate and amount varies depending on the AgNP characteristics determined by the size, stabilization and the presence of other molecules [17]. Still, some studies have discussed that AgNP toxicity cannot be explained solely by the release of Ag^+^ ions into the system [18–19]. Studies on AuNP toxicity provide conflicting results, with some authors claiming no toxicity was found [20–25], while others reported adverse effects caused by AuNPs [26–32]. Most likely, a mechanism of their toxicity is the generation of reactive oxygen species (ROS) and reactive nitrogen species (RNS) that trigger necrosis or apoptosis [33]. So far, a general consensus regarding NP toxicity is that their toxic effects cannot be conclusively defined, as the mechanism of their action depends on their physico-chemical properties (shape, size, charge, coating agents), but also on the targeted cells, tissues and/or organisms as well as on the type of testing itself [34–38]. Therefore, each individual NP type must be tested on various cell cultures using standardized and reliable assay protocols to adequately assess and explain the effects [39]. Exposure to NPs may lead to their uptake, translocation, and most likely, biotransformation within the body.

It has been demonstrated that AgNPs and AuNPs can be absorbed into systemic circulation via different routes [40–43]. Absorbed or intravenously applied NPs will accumulate in different body compartments [44–46]. AgNPs are prone to oxidative dissolution in biological media and the released Ag^+^ reacts with thiolate groups of sulfur-containing biomolecules [47], which may lead to the formation of thiol-coated AgNPs [48]. New clusters consisting of Ag_2_S can be created from free Ag^+^ [47]. This process, known as sulfidation, affects AgNP behavior in the biological system [49], most notably by reducing their toxicity [47,50]. The binding of thiols to AuNP surfaces has been well investigated [51–57]. As the most abundant intracellular biothiols are cysteine (CYS) and glutathione (GSH), their role in intracellular sulfidation and/or adsorption on the surface of NPs should be considered during the evaluation of efficacy, safety and behavior of AgNPs and AuNPs in vivo [47,58]. The biological effects of GSH-coated AuNPs and the potential for their biomedical use have been assessed [55–57]. However, there are still many knowledge gaps regarding the interaction between metal-based NPs and biothiols. The WoS search revealed less than 5% of the papers were related to studies on the nano–bio interface between biothiols and AgNPs or AuNPs (Figure S1 in Supporting Information File 1).

To gain further insight into the effect of biothiols on safety or toxicity of bioactive metallic NPs, this study aims to comprehensively investigate the mechanism of formation of AgNPs and AuNPs in the presence of CYS and GSH and their subsequent toxicity effects. Murine fibroblast L929 cells were selected as the in vitro model to study mammalian toxicity due to their properties. This cell line is non-cancerous, spontaneously immortalized, and one of the oldest continuous cell lines that can be properly maintained for a long time without being transformed. *Daphnia magna* was used as the representative aquatic organism model to evaluate ecotoxicity.

The comparison of the observed results with data published on other AgNP and AuNP types, as well as the results obtained for ionic Ag and Au forms, allowed for a better understanding of the role of CYS and GSH in biotransformation and biological effects of AgNPs and AuNPs. This study provided additional information to overcome knowledge gaps related to the design of safe and efficient AgNPs and AuNPs for biomedical uses.

## Results and Discussion

### Synthesis and characterization of nanoparticles

For the synthesis of AgNPs and AuNPs, a common bottom-up approach was applied using sodium borohydride as an agent to reduce Ag^+^ and Au^3+^, respectively. The optimization of the synthetic protocol was achieved by a series of experiments in which the reaction conditions (i.e., temperature, mixing time and rate, concentration of reactants) were carefully tested in order to obtain small, spherical and stable NPs. The molar ratio of the reactants [metallic salts]/[NaBH_4_]/[biothiol] were varied as summarized in Tables S1 and S2 of Supporting Information File 1. The particles were deemed unstable if they fully precipitated after synthesis and could not be redispersed, otherwise they were noted as stable. After each synthesis, the careful characterization of stable NPs was performed by dynamic light scattering (DLS), electrophoretic light scattering (ELS) and transmission electron microscopy (TEM) techniques. The obtained results showed that the molar ratio of reactants was more important for the successful preparation of stable NPs than all other parameters and experimental conditions. With regard to using CYS as a stabilizing agent, the results indicated that its molar concentration should be approximately ten times lower than the concentration of NaBH_4_, while GSH allowed for much wider concentration ranges. In the case of molar ratio reducing agent vs metallic salts, a larger excess of NaBH_4_ (>5 times) was needed to obtain stable NPs. Finally, we selected a molar ratio of [metallic salts]/[NaBH_4_]/[biothiol] = 1:10:1 for further work as it resulted in a favorable NP size (≈10 nm) and long term stability.

Nuclear magnetic resonance (NMR), as an excellent tool to determine the interactions of small organic molecules with metallic NPs, was applied to evaluate small changes in the chemical environment around NPs, which resulted in chemical shifts in the NMR spectra. Several studies have been published that confirm the validity of this technique in confirming thiol–NP interactions [51,59–61].

**Figure 1:**
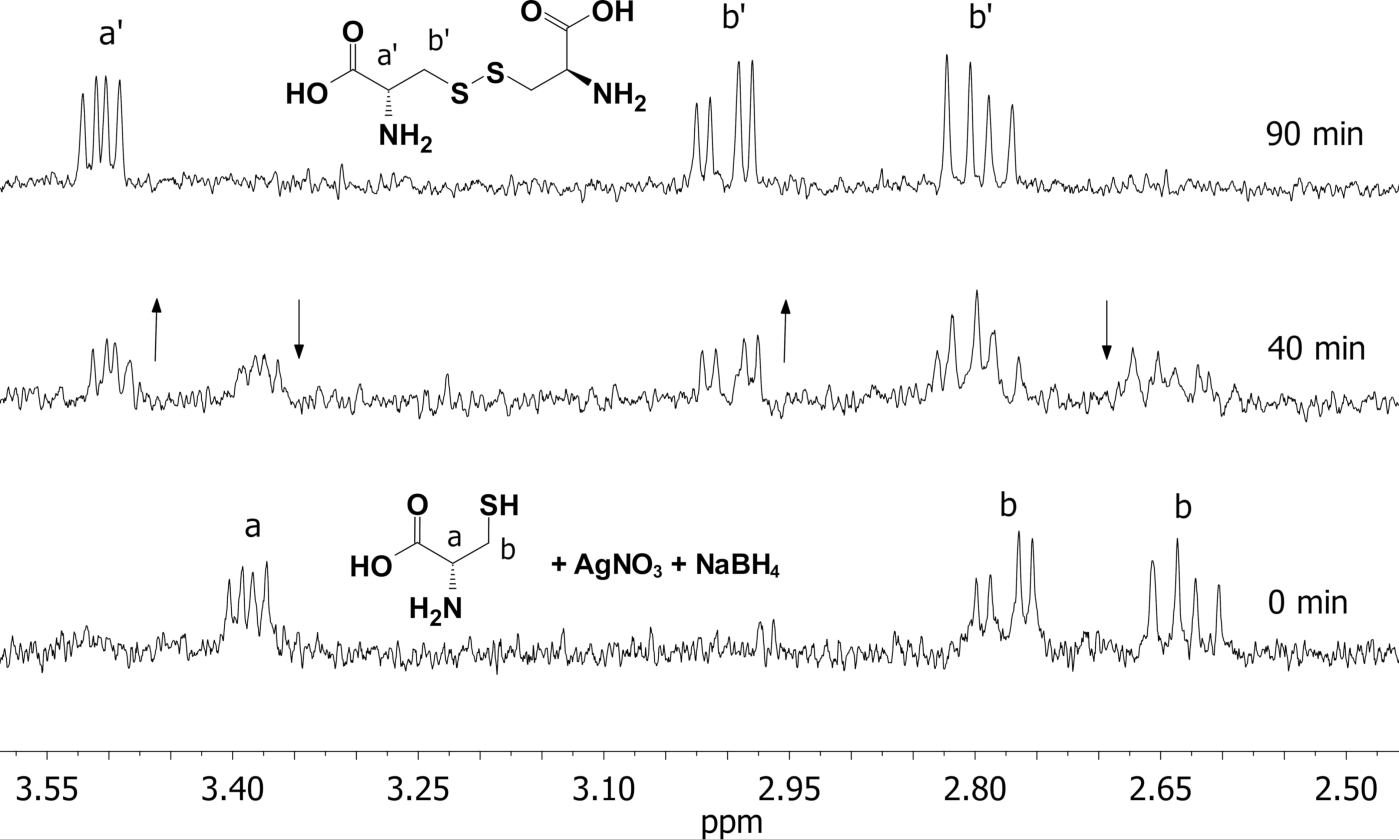
^1^H NMR spectra of the reaction mixture aliquots (5.6 mM cysteine, 56 mM NaBH_4_, and 5.6 mM AgNO_3_, in ultrapure water/D_2_O added) taken at several time points. The arrows show how proton signals (for cysteine and cystine) change with time.

In a typical experiment, the mixture of a metallic salt (HAuCl_4_ or AgNO_3_), NaBH_4_, and CYS (as described above) was stirred under argon in ultrapure water at room temperature for 90 minutes. The progress of the reaction was monitored by ^1^H NMR spectroscopy. Aliquots were taken from the reaction mixture at selected time points and D_2_O was added (or a D_2_O-filled capillary was used for a lock signal). Along with the disappearance of the ^1^H NMR signals of the reactant (CYS), a new set of proton signals of the product emerged (Figure 1). All new signals were shifted downfield by approximately 0.2 ppm. According to detailed ^1^H and ^13^C NMR analysis (see Figures S2–S4 in Supporting Information File 1), we conclude that cystine was formed.

After completion (no CYS signals in ^1^H NMR spectrum) the remaining signals were broadened in time, which indicates the binding of cystine to the NP surface. It is well known that ^1^H NMR signals from ligands bound to NPs display broad line widths [62–64].

Exactly the same ^1^H NMR profile was recorded for the reaction mixture which consisted of cysteine and NaBH_4_, i.e., no metallic salt was added (see Figure S4 in Supporting Information File 1). This means that during the regular procedure for the cysteine-coated NPs synthesis (as described elsewhere), the cystine may be formed prior to its binding to the NP surface. It was shown earlier that cysteine in the reaction with metallic salt (HAuCl_4_) underwent dimerization to form cysteine [65]. However, no NMR evidence was provided for that. In our case, the dimerization occurred in the reaction mixture consisting of NaBH_4_ and cysteine. It is possible that some NaBH_4_ hydrolysis products [66–68] induce this dimerization, but the detailed mechanism underlying the reaction was out of the scope of this paper.

**Figure 2:**
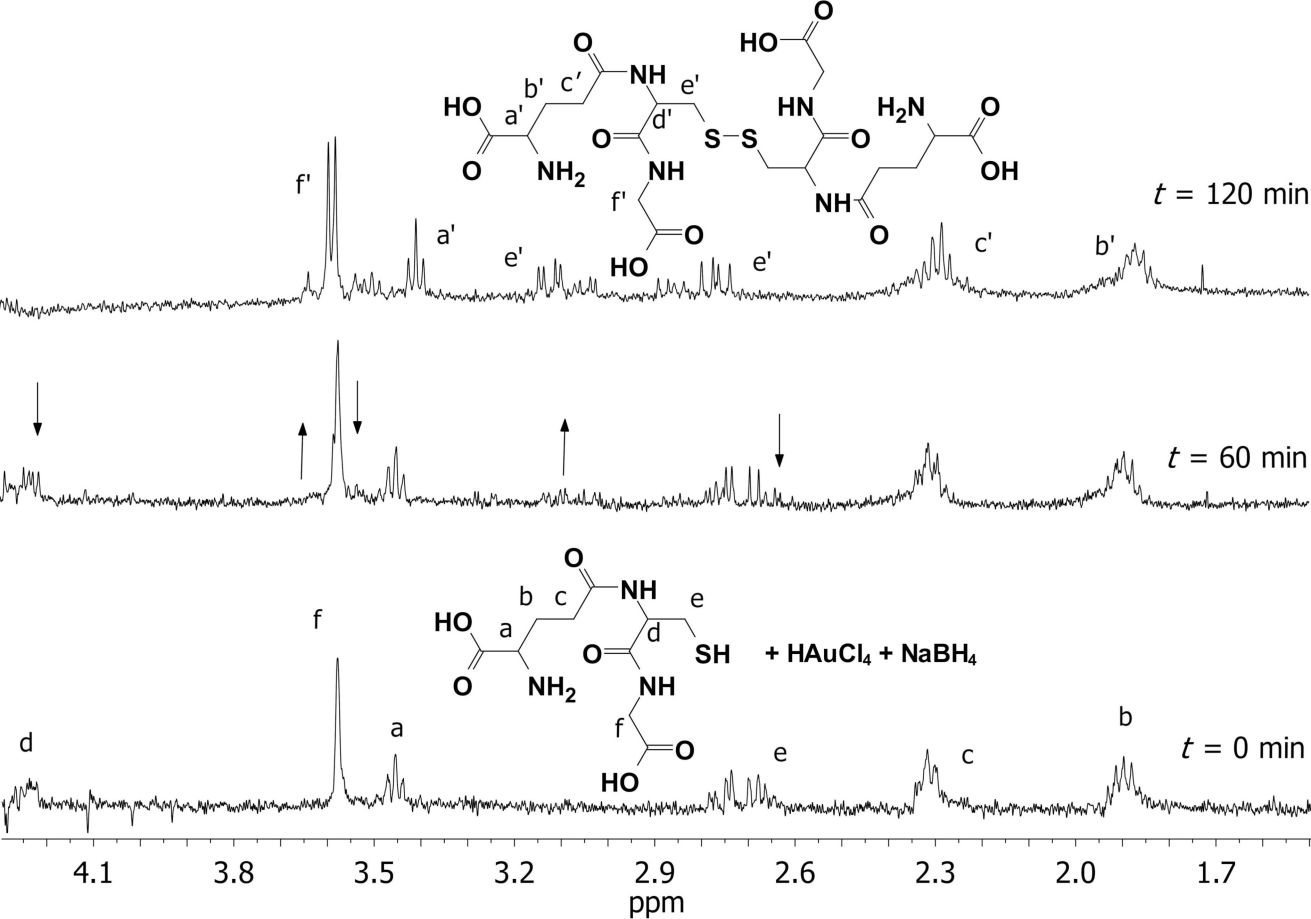
^1^H NMR spectra of the reaction mixture aliquots (5.6 mM glutathione, 56 mM NaBH_4_, and 5.6 mM HAuCl_4_, in ultrapure water/D_2_O added) taken at several time points. The arrows show how proton signals (for GSH and GSSG) change with time.

The very similar result was observed in the case of GSH which undergoes dimerization to GSSG in the presence of Au^3+^ (or Ag^+^) salt and NaBH_4_ (a standard protocol for the NPs synthesis) (Figure 2), or in the presence of NaBH_4_ alone (see Figure S5 in Supporting Information File 1). In conclusion, this indicates that both biothiols (GSH and CYS) bind to the NP surfaces in their oxidized form, which supports some earlier reports [51,69].

**Figure 3:**
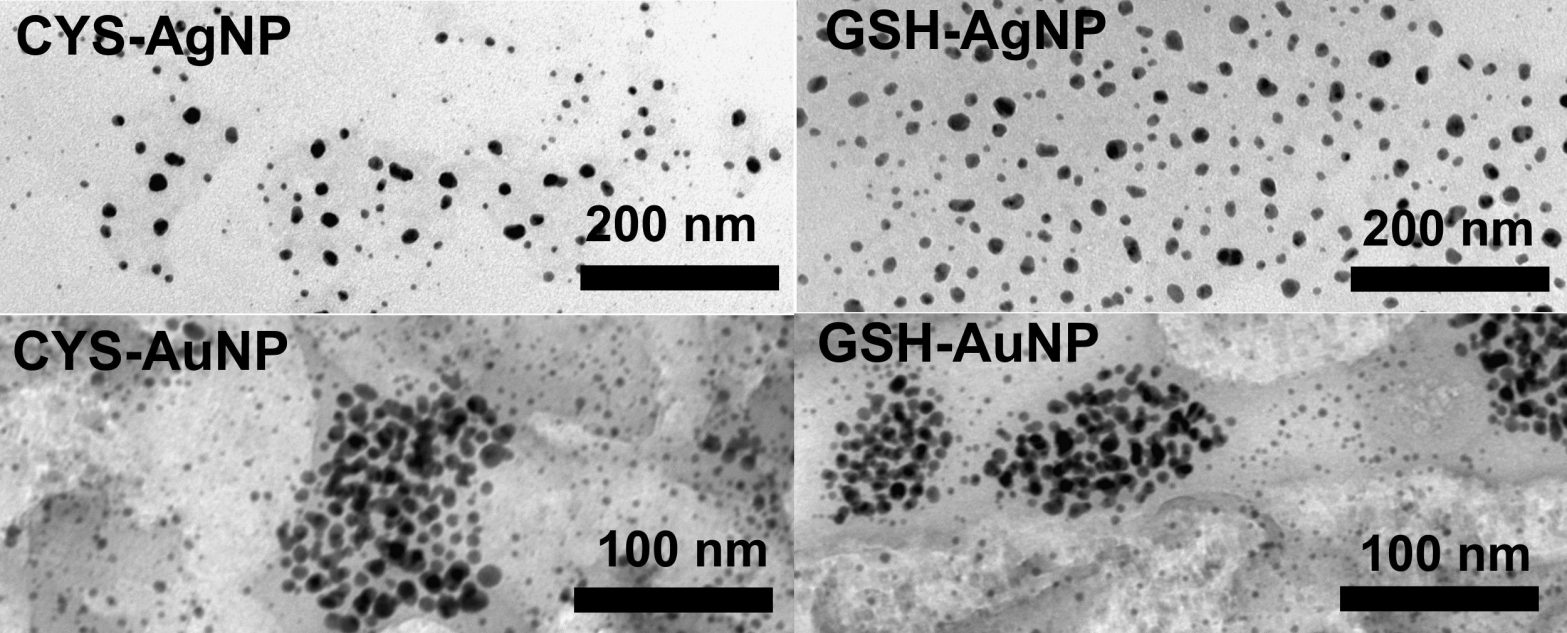
Transmission electron micrographs of silver (AgNPs) and gold nanoparticles (AuNPs) prepared in the presence of cysteine (CYS) or glutathione (GSH).

A detailed characterization of the prepared NPs dispersed in ultrapure water (UPW) revealed a negative surface charge. The observed zeta-potential values (Table 1) were indicative of a high NP stability, as the NPs are generally considered electrostatically stabilized when the absolute values of the zeta potential exceed 30 mV [70]. The size distribution of all NPs was bimodal and the AgNPs were generally smaller than the AuNPs (Table 1). TEM experiments showed a spherical shape for all NPs (Figure 3).

**Table 1:** Hydrodynamic diameter (*d*_H_) and zeta potential (ζ) of silver (AgNPs) and gold (AuNPs) nanoparticles stabilized with cysteine (CYS) or glutathione (GSH). The dissolution behavior was evaluated in ultrapure water (UPW), the cell culture medium used for L929 cells (EMEM) and the medium used for cultivation of *Daphnia magna* (SCM) after 24 h.

Nanoparticles	*d*_H_ [nm] (% mean volume)	ζ [mV]	% free metal ions		
			UPW	EMEM	SCM

CYS AuNPs	10.6 ± 2.4 (41%) 36.3 ± 8.6 (59%)	−46.8 ± 2.1	no dissolution		
CYS AgNPs	8.0 ± 0.9 (100%)	−56.9 ± 7.5	0.13	0.22	0.26
GSH AuNPs	7.4 ± 2.1 (38%) 65.1 ± 13.8 (62%)	−58.0 ± 3.8	no dissolution		
GSH AgNPs	6.0 ± 1.2 (100%)	−50.9 ± 2.3	0.10	0.77	0.64

As the biological effect resulting from interaction with metallic NPs may originate from the release of metallic ions in the biological environment, it was important to evaluate the dissolution behavior of the NPs. The release of Ag^+^ and Au^3+^ ions from AgNPs and AuNPs surfaces was studied in UPW and media relevant for biological experiments (i.e., EMEM + 10% FBS used as a culture media for fibroblasts and standard culture media (SCM) used for *D. magna*). As expected, the AuNPs did not dissolve in any of the tested media, while both CYS- and GSH-coated AgNPs released more ions in the media used for biological experiments (EMEM + 10% FBS and SCM) compared to UPW (Table 1). However, the amount of free Ag^+^ measured after incubation of AgNPs in these media for 24 h did not exceed 1% of total Ag. This does not necessarily mean that the Ag^+^ release is negligible, since underestimation of ionic Ag could happen due to complexes formed with components of the tested media that can precipitate or cannot pass the filter.

### Safety assessment

The toxicity of CYS- and GSH-coated AgNPs and AuNPs on mammalian cells was investigated in vitro to determine if biothiol-functionalization of metallic NPs reduces or increases their safety. For this purpose, the efficiency of NP uptake, cell viability, apoptosis induction, oxidative stress response and genotoxicity parameters of L929 cells treated with prepared NPs were determined and compared with control cells.

**Figure 4:**
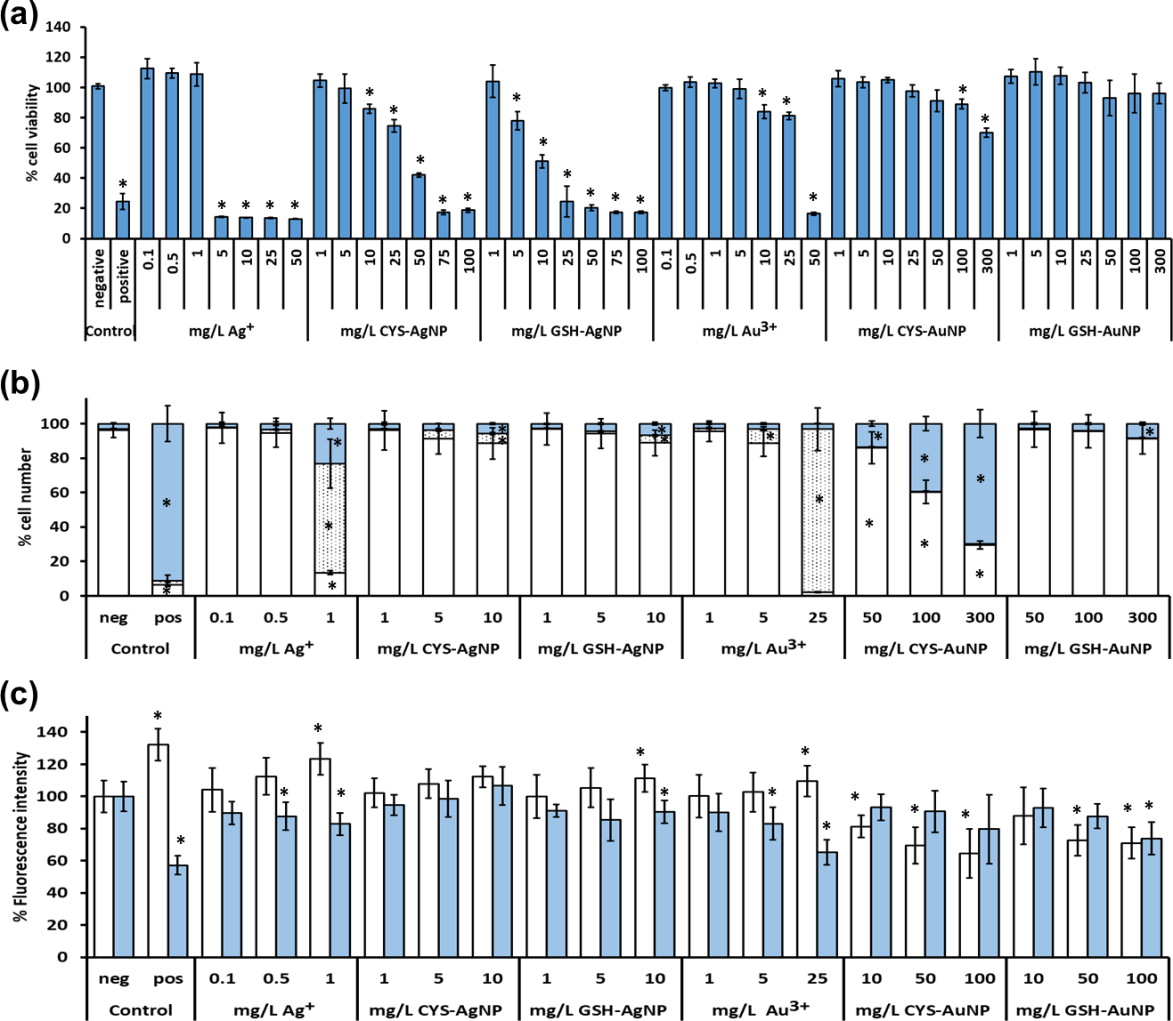
(a) Viability of L929 cells exposed to silver (AgNPs) and gold nanoparticles (AuNPs) stabilized with cysteine (CYS) or glutathione (GSH), Ag^+^ and Au^3+^ ions during 24 h and determined by the MTT cytotoxicity assay. (b) The effect of AgNPs, AuNPs, Ag^+^, and Au^3+^ on the number of live (white columns), early apoptotic (dotted columns) and late apoptotic (blue columns) L929 cells after 24 h exposure, determined by flow cytometry after Annexin V/PI staining. (c) The effect of AgNPs, AuNPs, Ag^+^, and Au^3+^ on the level of peroxy radical (white columns, as measured by DCFH-DA assay) and total GSH (blue columns, as measured by MBCl assay) in L929 cells after 4 h of exposure. Negative controls (neg) were untreated cells. Positive controls (pos) were cells treated with DMSO for MTT and Annexin V/PI assays, or with the *t*-butylhydroxyde for DCFH and MBCl assays. The results are expressed as percentage of negative controls and given as mean values obtained from three independent experiments. Standard deviations are presented as scale bars. Values marked with asterisk (*) differ significantly from the negative control (*P* < 0.05).

A range of NP concentrations were tested for negative effects on the survival and viability of L929 cells after 24 h exposure. Additional experiments included treatment of cells with different concentrations of Ag^+^ (in the form of AgNO_3_) and Au^3+^ (applied as HAuCl_4_) to determine if toxicity effects originate from the released counter ions. The results, as presented in Figure 4, demonstrated dose-dependent toxic effects of CYS- and GSH-coated AgNPs, while AuNPs showed no toxicity in the tested concentration range (1–300 mg Au L^−1^). However, GSH-coated AuNPs decreased cell viability by 20% at a dose of 300 mg Au L^−1^.

The GSH-coated AgNPs decreased cell viability by more than 50% at a dose 5 times lower than CYS-coated AgNPs. Ionic silver was the most toxic and destroyed the cells at 10 times higher dose compared to Au^3+^ (Figure 4a). Evidently, the most lethal tested substance was Ag^+^ followed by GSH-coated AgNPs. If we compare these results with data published on polymer-coated AgNPs and AuNPs (see Table S3 in Supporting Information File 1), the toxicity of GSH-AgNPs is similar to the toxicity effect of polyvinylpyrrolidone (PVP) stabilized AgNPs on the same cell type as reported earlier [71–72]. The PVP-coated NPs were selected for comparison as PVP is one of the most frequently used coating materials for stabilization of AuNPs and AgNPs [73]. Our results on AuNP cellular toxicity corroborate well with a similar study on L929 cells treated with PVP-coated AuNPs [74].

Dissolution experiments revealed that GSH-coated AgNPs at a concentration of 25 mg Ag L^−1^ would release only 0.2 mg Ag^+^ L^−1^ in the cell culture media, the concentration of ionic Ag that is non-toxic to L929 cells. Thus, the toxicity mechanism is much more complicated than a simple metal ion release in cell culture media. The cellular internalization of NPs by active transport may lead to intracellular NP dissolution, which may trigger a cascade of different toxic actions.

**Figure 5:**
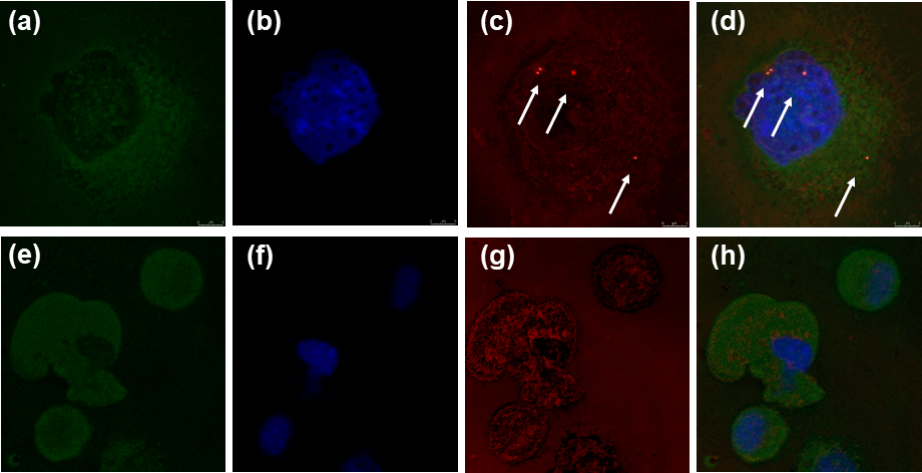
The NP uptake within L929 cells (a–d) compared to untreated control cells (e–h) as visualized by reflection mode confocal laser scanning microscopy (CLSM). The images show maximum intensity Z-projections of cells stained with phalloidin to stain actin and visualize cell cytoskeleton (green), nucleic acid staining using Hoechst 33258 fluorescent dye (blue) and CLSM reflectance signals (red). The overlay of fluorescence stains and segmented reflectance signals are given in (g,h). The control cells show no high intensity reflective spots (g), while NP reflectance signals are visible as bright red signals and indicated by white arrows (c,d).

Further safety tests were performed using a concentration range of NPs or ions that did not cause a decrease of cell viability by more than 80%. Uptake of all tested NPs, as determined by flow cytometry, showed dose-dependent cell penetration except for the GSH-AuNPs (Figure S6 in Supporting Information File 1). The highest efficiency of cell uptake was observed for CYS-coated AuNPs. For both CYS- and GSH-coated AgNPs, the uptake was significant only at the highest examined concentration (25 mg Ag L^−1^). The behavior of GSH AuNPs deviates considerably from the others and did not show any significant change in the side scattered light (SSC) ratio even at the highest dose applied, indicating no cell accumulation. The flow cytometry results on NP uptake were confirmed by visualization performed using confocal laser scanning microscopy (CLSM) in the reflection contrast mode. In cells treated with CYS-AgNPs, GSH-AgNPs and CYS-AuNPs, accumulated NPs were clearly visible intracellularly, while it was not possible to detect such accumulation in the case of treatment with GSH-AuNPs (see Figure 5 and Figure S7 in Supporting Information File 1). This is in accordance with recently published in vivo data on GSH-coated gold nanoclusters with promising theranostic properties [55–56]. Our GSH-AuNPs bypassed internalization by L929 cells similar to the interaction of GSH-protected Au nanoclusters with the reticuloendothelial system (RES) observed in these studies, which demonstrated that GSH played a protective role for AuNP-based theranostic systems against RES accumulation. Moreover, the conducted pharmacokinetic studies revealed that the use of GSH for the preparation of small AuNPs enhance tumor retention time, but also increased normal tissue clearance [55–56]. However, our results indicated a completely different interaction of CYS-AuNPs with non-cancerous L929 cells. The cell uptake clearly followed a dose-response pattern (Figure S6 in Supporting Information File 1), while confocal imaging showed the accumulation of CYS-AuNPs even in the cell nucleus (Figure 5), which can explain their genotoxicity and apoptosis induction ability (see discussion below).

To gain better insight into the toxicity mechanism of silver and gold NPs and their counter ions, the percentage of live, early apoptotic and late apoptotic/dead cells were determined by flow cytometry analysis after staining with CAM and EthD dyes. The doses that caused low or no significant reduction in cell viability (Figure 4a) showed apoptotic processes in a dose-response manner for all tested species (Figure 4b). In accordance with the MTT data (Figure 4a), ionic species triggered apoptosis in 86% and 95% of cells at the highest concentrations applied, i.e., 1 mg Ag L^−1^ and 25 mg Au L^−1^, respectively. Silver and gold NPs were safer, inducing at tested doses less than 20% of apoptotic events (Figure 4b). However, treatment with GSH-AuNPs led to a significant number of late apoptotic cells, while CYS- and GSH-coated AgNPs were shown to be safer than expected. In both cases, more than 88% of fibroblasts survived the treatment with the highest dose applied (10 mg Ag L^−1^).

As the exposure to NPs is known to induce oxidative stress in cells [75–76], ROS production and intracellular GSH levels were measured after 24 h incubation with the tested species. The results corroborated well with those on the number of apoptotic cells. In all cases where doses of tested induced apoptosis in fibroblasts, ROS levels were also significantly changed (Figure 4c). The ROS level was significantly increased after treatment with AgNPs and ionic metal species, but significantly decreased in the AuNP-treated cells, possibly due to more the effective activation of protective mechanisms against oxidative stress under these exposure scenarios [77]. A previous study on human pulmonary fibroblasts also found that AuNPs cause higher oxidative stress than AgNPs [78]. The level of GSH changed significantly compared to the control only in cases where the number of apoptotic cells was higher than 40%, i.e., cells treated with Au^3+^ or Ag^+^. Once more, the NPs were proven to be less toxic than their ionic counterparts, requiring 10–50 times higher doses to achieve the same toxic effect.

**Figure 6:**
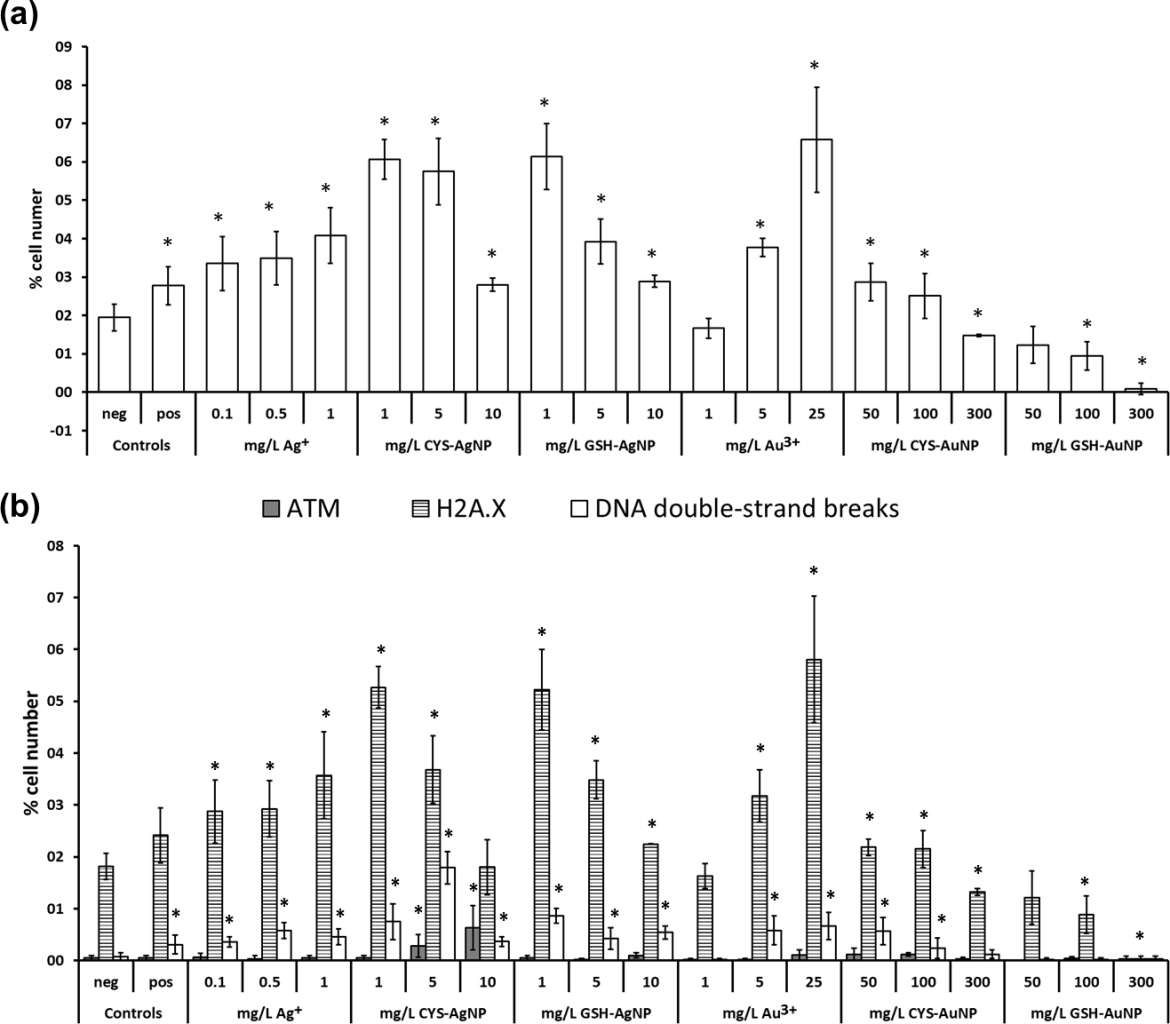
The effect of silver (AgNPs) and gold nanoparticles (AuNPs) stabilized with cysteine (CYS) or glutathione (GSH), Ag^+^ and Au^3+^ ions on (a) total DNA damage and (b) DNA double-stranded breaks (white columns), the activation of ATM (grey columns) and H2A.X (striped columns) in L929 cells after 24 h exposure, measured with Muse^TM^ Multi-Color DNA Damage Kit. Negative controls (Neg) were untreated cells, while positive controls (pos) were cells treated with DMSO. The results are expressed as the percentage of negative controls and given as mean values obtained from three independent experiments. Standard deviations are presented as scale bars. The values marked with an asterisk (*) differ significantly from the negative control (*P* < 0.05).

As oxidative stress may induce damage to DNA molecules [79], which will consequently lead to apoptosis if severe enough, the DNA damage signaling pathway was evaluated by detecting the ataxia–telangiectasia mutated (ATM) and H2A histone (H2A.X) activated cells, as well as their dual activation which indicates DNA double-strand breaks. DNA double-strand breaks may lead to chromosome aberrations, genomic instability, or cell death [80]. The evaluation of ATM and H2A.X activation is more accurate and is a quantitative measurement of DNA damage response at the single cell level. The total percentage of damaged cells is a sum of ATM positive, H2A.X positive and double-positive cells. Although the number of cells positive for DNA damage was low (Figure 6), a clear dose-dependent activation for each target was observed. In the case of ionic forms, the number of cells with DNA damage increased with the increase of Au^3+^ or Ag^+^ concentration, while opposite trends were observed in cells treated with NPs (Figure 6).

This could be the result of the higher mortality of the cells exposed to larger concentrations of AgNPs and AuNPs, lowering the overall number of cells with activated ATM and H2A.X. It is important to note that the total percentage of cells with DNA damage remains below 7% in all of the cases. Thus, the overall harmful effect of AgNPs and AuNPs on DNA was relatively small for the tested concentration range and duration of exposure (24 h), as evidenced also by evaluation of apoptosis and oxidative stress response.

In general, ionic species were found to be the most toxic, followed by AgNPs, while AuNPs can be considered safe to cells. With regard to the effects of the coating, the results are inconclusive. The GSH-AgNPs decreased cell viability at a lower concentration than CYS-AgNPs, but there were no significant differences between these two treatments for the induction of apoptosis, DNA damage or oxidative stress response. In the case of AuNPs, CYS-AuNPs were slightly more toxic than GSH-AgNPs with regard to cell viability, the number of cells in late apoptosis, and DNA damage, which can be explained by efficient internalization of these type of AuNPs (Figure 5, Figures S6 and S7 in Supporting Information File 1). The GSH-AuNPs proved to be not only less toxic than CYS-AuNPs, but even demonstrated lower activation of ATM and H2A.X than in control cells. However, the detailed mechanism behind these results should be investigated in future studies.

When comparing the toxicity of CYS- or GSH-coated NPs with those of the same core shell (Au or Ag) but coated with other types of stabilization agents, it may be concluded that CYS-AuNPs and GSH-AuNPs demonstrated low or even no toxicity as other types of AuNPs [74]. The toxicity of CYS-AgNPs was similar to other types of AgNPs [71], while GSH-AgNPs were demonstrated to be the most toxic type of AgNPs. However, this comparison only took into account the results published on the L929 cell line (Table S3 in Supporting Information File 1). As AuNPs demonstrated to be resistant to dissolution behavior in cell culture media, the increased toxicity of GSH-AgNPs may originate from the possible catalytic role of GSH on the dissolution process on the AgNP surface. Indeed, GSH-AgNPs released the highest amount of metallic ions in the culture media (Table 1), while additional dissolution may also be expected in endosomes following cell uptake.

### Acute toxicity test on *D. magna*

The ecotoxicological impact of CYS- and GSH-coated AuNPs and AgNPs was tested using aquatic crustacean *D. magna* Straus as a model organism due to its ecological significance and widespread use in regulatory testing [81]. Acute toxicity tests were performed after 24 and 48 h of exposure to NPs, while treatment with Ag^+^ and Au^3+^ was included for comparative purposes (Table 2).

**Table 2:** Acute toxicity of ionic silver and gold, silver (AgNPs) and gold nanoparticles (AuNPs) stabilized with cysteine (CYS) or glutathione (GSH), to *Daphnia magna* Straus. EC_50_ values, no adverse observed adverse effect levels (NOAEL) and lowest observed adverse effect levels (LOAEL) are given in µg Ag or Au L^−1^ and expressed as mean values obtained from 6 experiments and include standard deviations, while 95% confidence intervals are given in parentheses.

Species examined	EC_50_ (95% CI)		NOAEL		LOAEL	
	24 h	48 h	24 h	48 h	24 h	48 h

CYS-AgNP	347.6 ± 5.39 (344.19–364.39)	193.1 ± 5.20 (192.73–206.39)	50	20	100	50
GSH-AgNP	152.5 ± 6.20 (150.76–166.14)	119.0 ± 5.79 (116.92–131.31)	50	12	75	50
CYS-AuNP	–	–	2500	250	5000	500
GSH-AuNP	–	–	75	25	100	50
Ag^+^	1.06 ± 5.69 (0–12.75)	0.97 ± 5.13 (0–11.74)	0.1	<0.1	0.5	0.1
Au^3+^	12.7 ± 6.23 (12.15–27.61)	10.4 ± 6.19 (8.63–24.01)	5	4	7	5

The obtained EC_50_, no adverse observed adverse effect levels (NOAEL) and lowest observed adverse effect levels (LOAEL) values demonstrated significantly higher toxicity to Ag^+^ and Au^3+^ compared to AgNPs. It was not possible to determine the EC_50_ value for AuNPs, as the NOAEL and LOAEL values reached g L^−1^ levels for both CYS- and GSH-coated AuNPs. The CYS-AgNPs were slightly less toxic to *D. magna* than GSH-AgNPs. Indeed, the trend for toxicity potential of the tested ionic and nanoparticulate forms of Au and Ag were similar for aquatic organism as for mammalian cells. However, the concentrations that caused harmful effects were much lower in aquatic toxicity tests.

**Figure 7:**
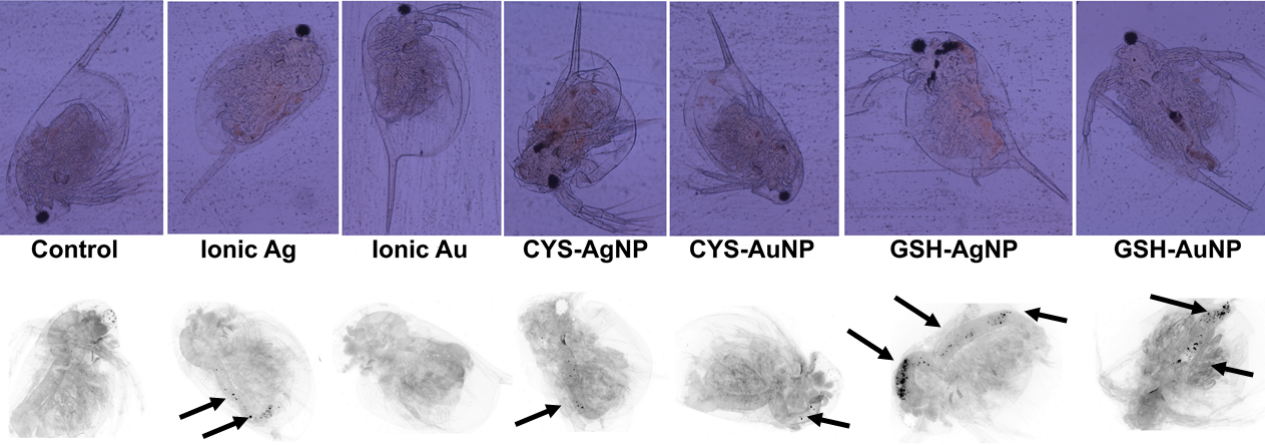
Micrographs of *D. magna* neonates after 48 h exposure to ionic and nanoparticulate forms of Ag and Au. Visible accumulation is marked with arrows.

The microscopic evaluation of treated and survived *D. magna* was additionally performed to assess if any accumulated NPs can be found in the digestive tract of these animals. Deposits of NPs, but also of ingested ionic Ag, were visible as black dots (Figure 7). No traces of Au^3+^ were found. These micrographs evidenced that *D. magna* was exposed to NPs through digestion. Such results manifest the harmful environmental effects of metallic NPs, as already evidenced in earlier studies [82–84].

The comparison of these results with previously published toxicity data on polymer- or citrate-coated AgNPs and AuNPs indicated that functionalization with CYS- or GSH-reduced the NP toxicity towards *D. magna* (see Table S4 in Supporting Information File 1).

## Conclusion

As the interest in metallic nanoparticles for biomedical use grows, the need emerges for the development of safe, surface-modified nanosystems. For the first time, safety profiling of AgNPs and AuNPs prepared by using cysteine and glutathione as stabilizing agents was performed. A careful and comprehensive evaluation of physico-chemical properties and surface modification of these NPs revealed that stabilization of both AgNPs and AuNPs occurred through interaction of the metallic nanosurface with disulfide of oxidized forms of cysteine and glutathione. This important observation sheds new light on the mechanism of NP formation in the presence of biothiols.

The in vivo assessment of NP toxicity on aquatic organisms evidenced that surface modification of metallic NPs with biothiols made them safer for the environment, while in vitro experiments on mammalian cells demonstrated that AgNPs and AuNPs functionalized with glutathione had a significant biological impact. An increased toxicity effect was observed for the AgNPs prepared in the presence of GSH, but a decreased toxicity was observed in the case of AuNPs. Further studies should be directed toward transformation patterns of NPs in vivo and the role of nano–bio interactions that may either lead to less or more toxic-transformed nanospecies.

## Materials and Methods

### Chemicals and reagents

All chemicals and materials were purchased from Sigma Aldrich (Darmstadt, Germany) unless stated otherwise. Silver nitrate (AgNO_3_) was purchased from Alfa Aesar (Karlsruhe, Germany). All compounds were reagent-grade or higher.

### Synthesis and characterization of AgNPs and AuNPs in the presence of CYS and GSH

AgNPs and AuNPs were synthesized by the reduction of silver and gold salts, respectively, using sodium borohydride in the presence of CYS or GSH as stabilizing agents. Several synthetic approaches were tested, where different experimental parameters were employed (concentration and ratio of reactants, ordering of reactants addition, mixing time and speed, etc.) as presented in Tables S1 and S2 given in Supporting Information. All syntheses were performed at room temperature and the mixtures were protected from light. The obtained NPs were carefully characterized by means of size distribution and surface charge employing dynamic light scattering (DLS) and electrophoretic light scattering (ELS) methods. The visualization of the NPs was performed using TEM. The most stable AuNPs and AgNPs with similar physico-chemical properties were obtained by a procedure in which appropriate amounts of HAuCl_4_ or AgNO_3_ were dissolved in ultrapure water (UPW). The biothiol solutions (CYS or GSH) were then added and stirred for 10 min. Finally, NaBH_4_ was added dropwise under vigorous stirring. The mixture was left to react for 2 h. The molar ratio of [metallic salt]/[reducing agent]/[biothiol] = 1:10:1 was chosen. For AuNPs, the final concentration of reagents was 3 mM HAuCl_4_, 30 mM NaBH_4_ and 3 mM biothiol, while 5.6 mM AgNO_3_, 56 mM NaBH_4_ and 5.6 mM biothiol were employed for AgNPs. After the synthesis, each of the NP solutions were washed twice with UPW using ultracentrifugation at 15000*g* for 40 min. The washed NPs were kept in the dark at 4 °C.

The concentration of AgNPs and AuNPs was determined as the total Ag and Au content, respectively, analyzed by an Agilent Technologies 7800 inductively coupled plasma mass spectrometer (ICPMS) (Agilent, Waldbronn, Germany). Visualization was performed using a TEM 902A instrument (Carl Zeiss Meditec AG, Jena, Germany). The microscope was operated in the bright-field mode and at an acceleration voltage of 80 kV. The TEM samples were prepared on a Formvar^®^-coated copper grid (SPI Supplies, West Chester, PA, USA) by depositing a drop of the particle suspension and leaving it to air-dry at room temperature. The images were recorded with an attached Canon PowerShot S50 camera. Both DLS and ELS measurements were performed at room temperature using a Zetasizer Nano ZS instrument (Malvern Instruments, Malvern, UK) with a green laser (532 nm), set at an angle of 173°. The size distribution of the NPs is expressed as the hydrodynamic diameter (*d*_H_) obtained from the size-volume distribution function and given as an average of 10 measurements. The surface charge was determined by measuring the electrophoretic mobility, which was converted to zeta potential (ζ) values using the Henry equation with the Smoluchowski approximation. The measurements were repeated five times. The DLS and ELS data processing was performed using Zetasizer software 6.32 (Malvern Instruments, Malvern, UK).

The dissolution behavior of AuNPs and AgNPs was tested by ultrafiltration followed by quantification of released free gold or silver ions. The test media were UPW, cell culture medium EMEM with the addition of 10% FBS, and standard culture media for *Daphnia magna* cultivation (SCM). Freshly prepared NPs were diluted in the test media to a final concentration of 10 mg L^−1^, and were left stirring in the dark for 1 h following filtration using an Amicon-4 Ultra centrifugal filter of 3 kDa cut-off size (Merck Millipore, Darmstadt, Germany). The obtained filtrates were immediately acidified with HNO_3_ to a final acid content of 10% (v/v). The released silver or gold ions were quantified by ICP-MS. The reliability of the employed analytical method was confirmed using the Standard Reference Material^®^ (SRM) 1643e Trace Elements in Water (NIST, Gaithersburg, Maryland, USA). The results are presented as average values of five independent measurements.

### NMR experiments

The progress of the reaction in the mixture of biothiols, metallic salts, and/or NaBH_4_, was followed by ^1^H and ^13^C NMR spectroscopy using a Varian INOVA 400 spectrometer (Varian, Palo Alto, CA, USA) operating at 399.6 and 99.9 MHz, respectively. The samples were prepared in UPW with the addition of D_2_O to a final volume ratio of 10%, or in some cases, a capillary filled with D_2_O was used as an external lock. ^1^H NMR spectra were recorded in 10–20 min intervals during the NP synthesis, starting immediately after the mixing of reagents (*t*_0_). The chemical shifts were expressed in parts per million (ppm) and are referenced to the residual water signal. All spectra were recorded at 25 °C. For all experiments, a recycle delay of 5 s was used, which was sufficiently greater than the relaxation time *T*_1_. To suppress the solvent signal, the WET and PRESAT pulse sequences were used, as available in VnmrJ (4.2A) software.

### Cell experiments

Murine fibroblast (NCTC clone L929) cells (ATCC^®^ CCL-1^TM^) were cultured in EMEM with the addition of FBS (10%) and penicillin/streptomycin (1%) in a T25 culture flask (Eppendorf, Hamburg, Germany). The cells were regularly tested for absence of mycoplasma by means of a direct DNA dye test [85]. When the cells reached 80% confluence, the culture medium was removed with a pipette; the cells were washed once with sterile phosphate buffer saline (PBS), detached from the flask by adding trypsin/EDTA (0.25%) solution and incubated for 10 min at 37 °C and 5% CO_2_. The detached cells were collected, counted on a TC20 automated cell counter (Biorad, Invine, CA, USA), and seeded in sterile 96-well plates or 12-well plates (Eppendorf, Hamburg, Germany) for subsequent treatment. The seeded cells were grown for 24 h at 37 °C and 5% CO_2_ to allow cell attachment. The following day, AgNPs, AuNPs, AgNO_3_ and HAuCl_4_ were added to the wells in different concentrations and treated for 24 h.

The MTT assay, based on the reduction of the yellow tetrazolium salt MTT (3-(4,5-dimethylthiazol-2-yl)-2,5-diphenyltetrazolium bromide) to a purple MTT-formazan crystal by metabolically active cells, was used to determine cell viability after 24 h exposure. The assay was performed according to the manufacturer’s instructions. Briefly, the cells were seeded in 96-well tissue culture plates (5 × 10^4^ cells/mL growth medium, i.e., 1.5 × 10^4^ cells/cm^2^) followed by an overnight incubation. The different concentrations of NPs or salt suspensions were added (to a final concentration range of 1–100 mg L^−1^ for AgNPs, 1–300 mg L^−1^ for AuNPs) and left to incubate for 24 h. For the purpose of comparison, the cells were also incubated with AgNO_3_ and HAuCl_4_, in the final concentration range of 0.1–50 mg L^−1^. The untreated cells were used as negative controls, while the cells treated with dimethyl sulfoxide (DMSO) (10% (v/v)) were used as positive controls. After incubation, the medium was removed from the wells by aspiration; the cells were washed three times with PBS (200 μL per well) and MTT solution (50 μL, 1000 mg L^−1^) was added to each well. The dye was left to incubate for 4 h at 37 °C, after which the MTT solution was removed by aspiration. The remaining formazan crystals were dissolved by addition of DMSO (50 μL) and shaking the plates. The absorbance was recorded at 530 nm using a Victor^TM^ multilabel reader (Perkin Elmer, Massachusetts, USA).

The ratio of live, apoptotic and dead cells after treatment with NPs or respective metal salts was determined by flow cytometry experiments using Annexin V and 7-aminoactinomycin D (7-AAD) staining. Annexin V binds phosphatidylserine, which can only be found on the outer leaflet of cell membranes during apoptosis, while 7-AAD is a DNA-binding agent that cannot penetrate the membrane of living cells and can only stain dead or late apoptotic ones. The treatment involved 24 h incubation of cells with AgNPs (ranging between 1–10 mg Ag L^−1^), AuNPs (50–300 mg Au L^−1^), AgNO_3_ (0.1–1 mg Ag L^−1^) and HAuCl_4_ (1–25 mg Au L^−1^) in 6-well plates at a density of 2.5 × 10^5^ cells/well, i.e., 2.6 × 10^5^ cells/cm^2^. Positive controls were cells treated with paraformaldehyde (0.04 mg L^−1^), while untreated cells were negative controls. After treatment, the plates were centrifuged at 1500*g* for 15 min. Supernatants containing dead cells were collected from 12-well plates in 1.5 mL Eppendorf tubes. Live cells were detached from the wells by adding 0.05 % GibcoTM trypsin/EDTA (Thermo Fisher Scientific, Waltham, USA) solution, washed with PBS, resuspended in PBS-based buffer containing FBS (2%) and EDTA (2 mmol L^−1^) (pH 7.4, filtered through 0,2 μm sterile filter) and passed through a 40 μm Falcon^TM^ cell strainer (Thermo Fisher Scientific, Waltham, USA). The supernatants and the detached cells were joined, centrifuged at 800*g* for 5 min and washed with PBS containing 2% bovine serum albumin (1 mL per sample). The cells were then stained using Muse^®^ Annexin V and a dead cell assay kit (Merck KGaA, Darmstadt, Germany) according to the manufacturer’s instructions. After staining, the cells were washed twice by adding PBS (1 mL per sample) and analyzed using a Muse^TM^ cell analyzer along with the corresponding Muse^TM^ software module. The data are expressed as percent relative to negative controls.

The internalization of the NPs was evaluated using a Molecular Probes^TM^ LIVE/DEAD^TM^ kit (Invitrogen, Thermo Fisher Scientific, Waltham, USA) and an Attune^®^ acoustic focusing flow cytometer (Applied Biosystems, Carlsbad, CA, USA) equipped with a 488 nm laser. For the experiment, L929 cells were seeded in 6-well plates at a density 2.5 × 10^5^ cells/well. After 48 h, the culture medium was exchanged with a fresh one and increasing concentrations of AgNPs (1, 5 or 25 mg Ag L^−1^) or AuNPs (25, 50 or 100 mg Au L^−1^) were added. Negative controls were non-treated cells. The dissociated cells were incubated with calcein acetoxymethyl ester (CAM, 0.1 µM) and ethidium homodimer-1 (EthD, 3 μM), both supplied in the kit, for 15 min in the dark at room temperature. Each experiment was repeated at least three times. CAM and EthD were measured using log amplifiers. The percentage of NP-labelled cells was determined using the Attune acoustic focusing cytometer by measuring the increase of the SSC of the laser beam. The intensity of the SSC is proportional to the intracellular density and granularity [86]. As NP uptake increases the intracellular density, the SSC intensity is also enhanced. The results were analyzed by FCS Express 5 Flow Cytometry Software using Overton the cumulative histogram subtraction method [87].

The reflection contrast mode of CLSM, as excellent non-invasive imaging strategy for label-free real-time tracking and quantification of non-fluorescent NPs [88], was used to visualize cellular uptake of NPs. In brief, the cells were grown in a 12-well chamber with glass slides (Eppendorf, Hamburg, Germany). After treatment with AgNPs or AuNPs (at 5 mg Ag/Au L^−1^), the cells were washed with PBS, fixed with ice-cold methanol (−20 °C), and stained with F-actin-phalloidin and Hoechst 33258 nucleic acid dyes. The slides were then mounted in Fluoroshield Antifade Mounting Medium and their images were recorded using a Leica TCS-SPE CLSM (Leica, Munich, Germany).

ROS production in cells treated with AgNPs and AuNPs was determined by the 2′,7′-dichlorodihydrofluorescein diacetate (DCFH-DA) staining. DCFH-DA is a non-fluorescent dye that can freely permeate the cell membrane. Inside the cell, it is hydrolyzed by cellular esterases to form DCFH, which is then oxidized by intracellular ROS to fluorescent 2′,7′-dichlorofluorescein (DCF). After treatment of L929 cells with AgNPs (in concentration range 1–5 mg Ag L^−1^), AuNPs (10–100 mg Au L^−1^), AgNO_3_ (0.1–1 mg Ag L^−1^) and HAuCl_4_ (1–25 mg Au L^−1^) for 4 h at 37 °C, the cells were washed three times with PBS and stained with DCFH-DA (20 μM) for 30 min at 37 °C. Then, the cells were washed again with PBS two times and analyzed using a Victor^TM^ multilabel reader (Perkin Elmer, Hopkinton, MA, USA) at an excitation wavelength of 485 nm and emission wavelength of 535 nm. The untreated cells were used as negative controls, while cells treated with hydrogen peroxide (100 μM) served as positive controls. The data are expressed as percentage of fluorescence intensity compared to negative controls.

The changes in the intracellular level of GSH were measured using monochlorobimane (MBCl) staining. This fluorogenic bimane probe forms a fluorescent adduct with GSH [89]. The treatment of the cells was performed following the same protocol as for the DCFH-DA assay, and the same controls were used. After treatment, the cells were washed three times with PBS, incubated with MBCl (50 μM) for 20 min at 37 °C, washed again twice with PBS and analyzed using a Victor^TM^ multilabel reader at an excitation wavelength of 355 nm and emission wavelength of 460 nm. The data are expressed as percentage of fluorescence intensity compared to negative controls.

DNA damage in L9292 cells was assessed using the Muse^TM^ Multi-Color DNA Damage Kit (EMD Millipore, Merck, Darmstadt, Germany) according to the manufacturer’s instructions. The kit measures the activation of ATM and H2A.X by phosphorylation, using phospho-specific ATM (Ser1981)-PE and phospho-specific histone H2A.X-PECy5 conjugated antibodies. This kit simultaneously provides statistical data for each of the two critical DNA damage markers at the single-cell level. The percentages of negative (undamaged) cells, ATM activated cells, H2A.X activated cells and DNA double strand breaks (dual activation of both ATM and H2A.X) were counted on the Muse^TM^ Cell Analyzer along with the corresponding Muse^TM^ software module.

Each cell experiment was repeated at least three times. The L929 cells were used between 10 and 18 passages for all experiments.

Additionally, the evaluation of interference between NPs and each assay was carried out for different concentrations of AgNPs and AuNPs (1, 10, 50 and 100 mg Ag L^−1^ or 1, 10, 50, 100 and 300 mg Au L^−1^, respectively) in cell-free culture medium following protocols as defined by the assay producers. The testing of interference with MTT, DCFH-DA and MBCl assays was carried out in 96-well plates using a Victor^TM^ plate reader following protocols as described previously [90]. As the interference of AuNPs and AgNPs with these assays stems exclusively from the optical properties of the NPs, interference was avoided by eliminating NPs from the test system using careful washing steps of the cells after treatment and before the staining procedures as indicated in the cell protocols.

In the case of flow cytometry experiments, the settings on the flow cytometer were carefully established taking into account testing of the correct NP concentration range, system noise, and evaluation of SSC signals that may be triggered by NPs. The highest concentration of each NP solution was run first to set the range for the maximum SSC signal. Gate settings for each experiment were performed using several different positive and negative controls, including NPs with and without dyes, unstained untreated cells, and stained untreated cell. Due to their small size, all AgNPs and AuNPs had a completely different scattering pattern than the L929 cells, and were easily gated out for elimination of background interference. To diminish interference that may arise from false signals of labelled NPs that could have been uptaken by live cells, we applied several washing steps to the NP-treated cells before the staining procedures.

### Acute toxicity test on *Daphnia magna*

Acute ecotoxicity tests were performed on *D. magna* Straus, an aquatic model organism for ecotoxicity tests. *D. magna* Straus clone MBP996 was purchased as Toxkit Ephippia (MicroBio-Tests Inc., Mariakerke, Belgium). Dormant eggs (ephippia) were incubated in standard culture media (SCM) prepared as reconstituted hard water with the addition of CaCl_2_·2H_2_O (294 mg L^−1^), MgSO_4_·7H_2_O (123.25 mg L^−1^), NaHCO_3_ (64.75 mg L^−1^), and KCl (5.75 mg L^−1^) at pH 7.8 ± 0.5, without any organic compounds. HRN EN ISO 6341:2013 protocol and Toxkit Ephippia supplier instructions for handling were followed. The exposure was carried out in the dark and the temperature was maintained between 19 and 21 °C.

The concentrated stock solutions of AgNPs, AuNPs, AgNO_3_ and HAuCl_4_ were diluted in the SCM to concentrations ranging between 5–1000 mg Ag L^−1^, 5–5000 mg Au L^−1^, 0.1–10 mg Ag L^−1^ and 0.5–50 mg Au L^−1^, respectively. The properties of the SCM such as temperature, pH, conductivity and oxygen levels were assessed using the respective probes from Orion Research Inc. (Jacksonville, FL, USA) at the start and end of the experiment. Groups of five *D. magna* neonates (24 h old) were transferred into glass test vessels that contained 10 mL of SCM (control) or tested compounds diluted in the SCM. Great care was taken during the transfer to avoid damaging any daphnids, which would lead to false conclusions. The daphnids were incubated for 24 and 48 h without feeding during the treatment period. The tests were performed four times each in four replicates. The death of the *D. magna* organism was the endpoint of the test. The acute toxicity was expressed as EC_50_, which is the effective concentration that causes mortality of 50% daphnids, with a 95% confidence interval (CI). The NOAEL and LOAEL were also recorded. In addition, surviving and normal daphnids were inspected by light microscopy to visualize the accumulation of Ag or Au in the gut. After 48 h of treatment, the daphnids were washed with PB pH 7.4 and put on microscopic glass using cover slips mounted with Vectashield (Vector Laboratories, Burlingame, CA, USA). An Opton III RS fluorescence microscope (Opton Feintechnik, Oberkochen, Germany) with ×10 magnification was used for examination and the images were recorded with a Spot RT Slider camera (Diagnostic Instruments, Sterling Heights, MI, USA). The image processing was performed in Adobe Photoshop 6.0.

### Statistical analysis

Statistical analysis was carried out using Dell^TM^ Statistica^TM^ 13.2 software (StatSoft, Tulsa, USA). The data represent mean values with standard deviations. The differences between treatments for the different measured variables were tested using simple and repeated measures ANOVA, followed by Fisher LSD post-hoc test when significant differences were found (*P* < 0.05). The homogeneity of variances was tested using the Levene test. The level of significance (*P* < 0.05) is indicated by the asterisks (*) for differences between treatments and controls.

## Supporting Information

File 1 Additional figures and tables.
