# Supplementary material for: Toxicity and safety study of silver and gold nanoparticles functionalized with cysteine and glutathione
Source: Beilstein J Nanotechnol. 2019 Sep 2;10:1802–17. doi: 10.3762/bjnano.10.175 (PMC6753685; doi:10.3762/bjnano.10.175)
Supplement: File 1 — Additional figures and tables. [file Beilstein_J_Nanotechnol-10-1802-s001.pdf]

BEILSTEIN JOURNAL OF NANOTECHNOLOGY

Supporting Information

for

Toxicity and safety study of silver and gold nanoparticles functionalized with cysteine and glutathione

Barbara Pem, Igor M. Pongrac, Lea Ulm, Ivan Pavičić, Valerije Vrček, Darija Domazet Jurašin, Marija Ljubojević, Adela Krivohlavek and Ivana Vinković Vrček

Beilstein J. Nanotechnol. **2019,** *10,* 1802–1817. doi:10.3762/bjnano.10.175

Additional figures and tables

License and Terms: This is a supporting information file under the terms of the Creative Commons Attribution License (http://creativecommons.org/licenses/by/4.0). Please note that the reuse, redistribution and reproduction in particular requires that the authors and source are credited. The license is subject to the *Beilstein Journal of Nanotechnology* terms and conditions: (https://www.beilstein-journals.org/bjnano)

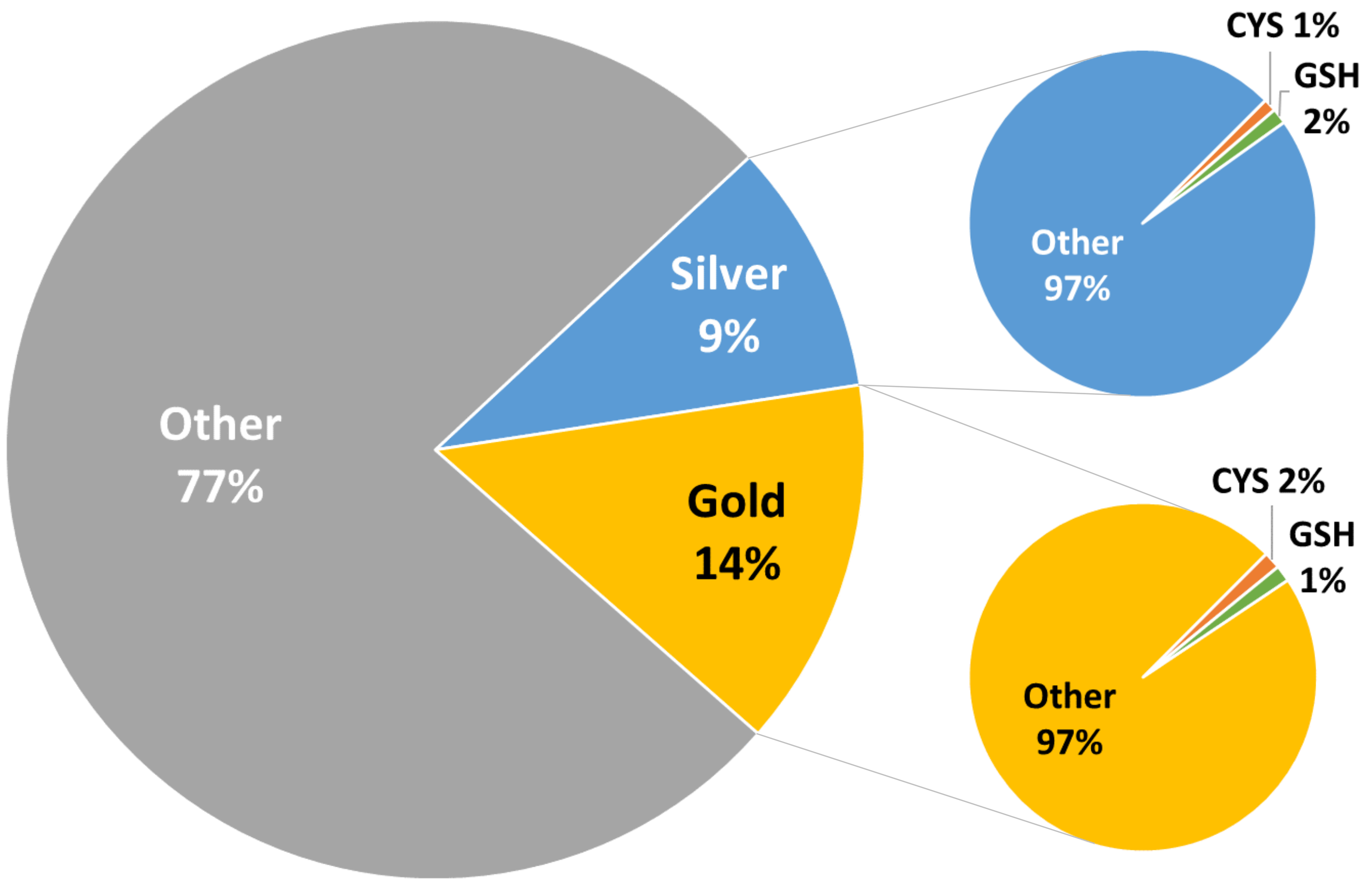

Figure S1: The percentage of research papers focusing on silver (AgNPs) and gold nanoparticles (AuNPs) out of the total amount of papers discussing nanoparticles for biomedical applications (larger chart). Two smaller charts present the percentage of papers on interaction of AgNPs and AuNPs with cysteine (CYS) or glutathione (GSH).

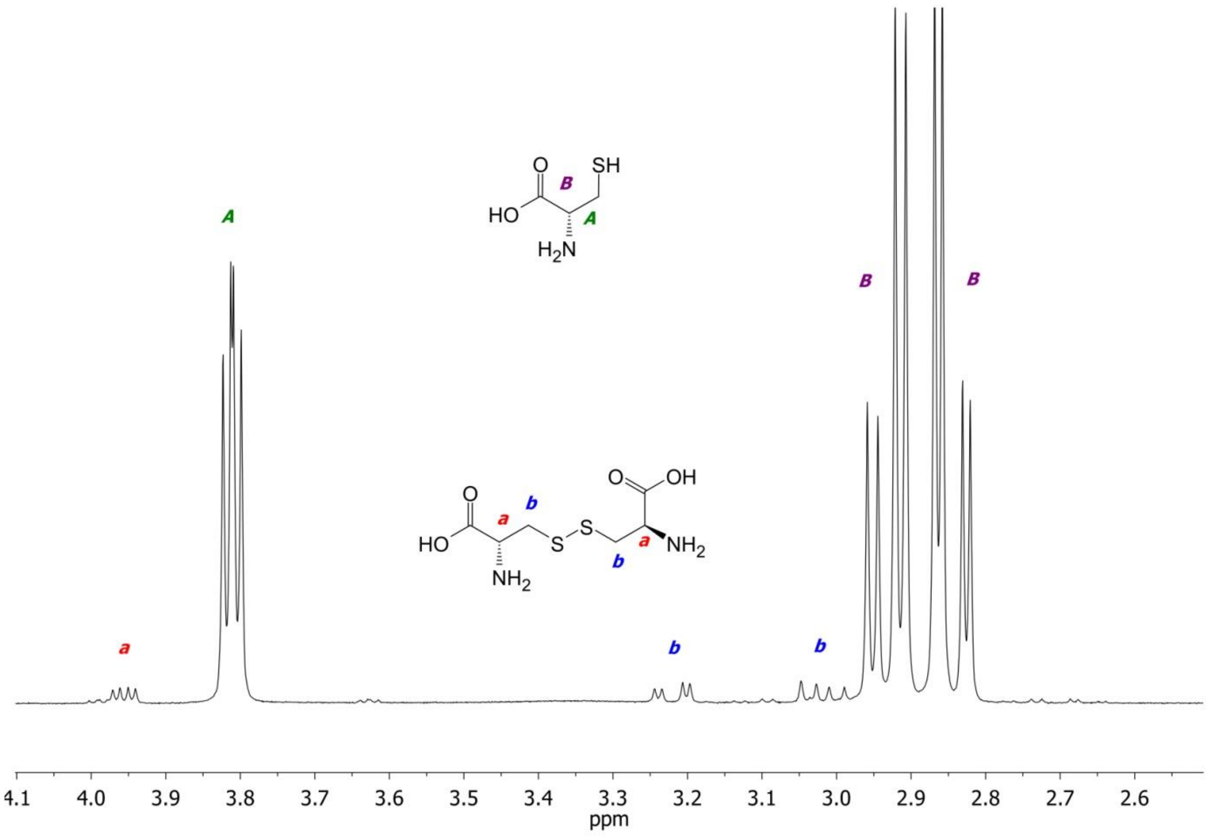

NMR analysis

Figure S2: ^{1}H NMR spectrum of the mixture cysteine : cystine = 25 : 1, in D_2O. Commercial samples (Sigma Aldrich) of both compounds were used.

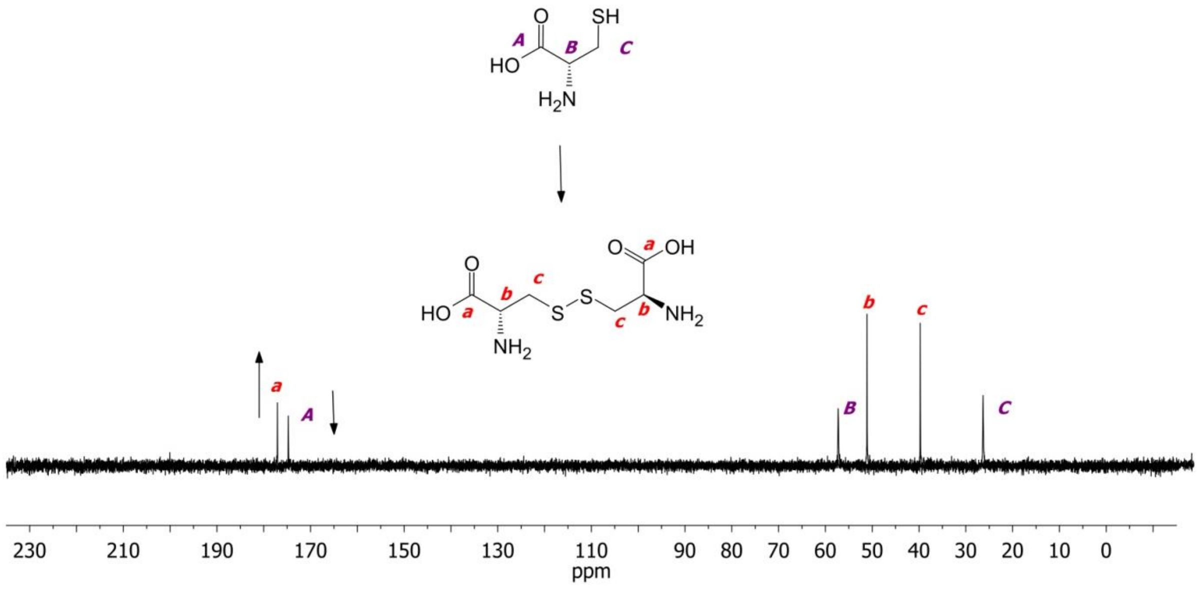

Figure S3: ^{13}C NMR spectrum of the reaction mixture cysteine : $NaBH_4$ = 1 : 10, in D_2O. The aliqout is taken after 75 min. The arrows show how signals (for cysteine and cystine) change with time.

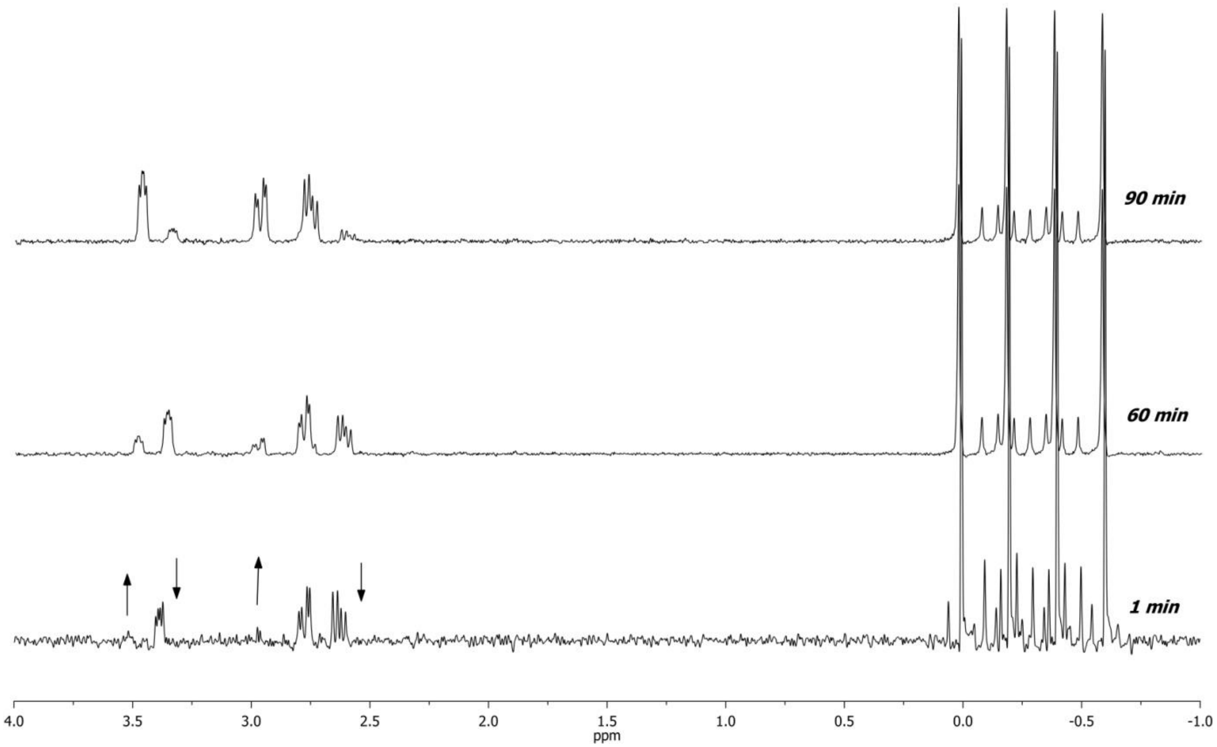

Figure S4: ^{1}H NMR spectra of the reaction mixture aliquots (cysteine : $NaBH_4$ = 1 : 10, in ultrapure water/D_2O added) taken at several time points. The arrows show how signals (for cysteine and cystine) change with time. Upfield signals correspond to $NaBH_4$.

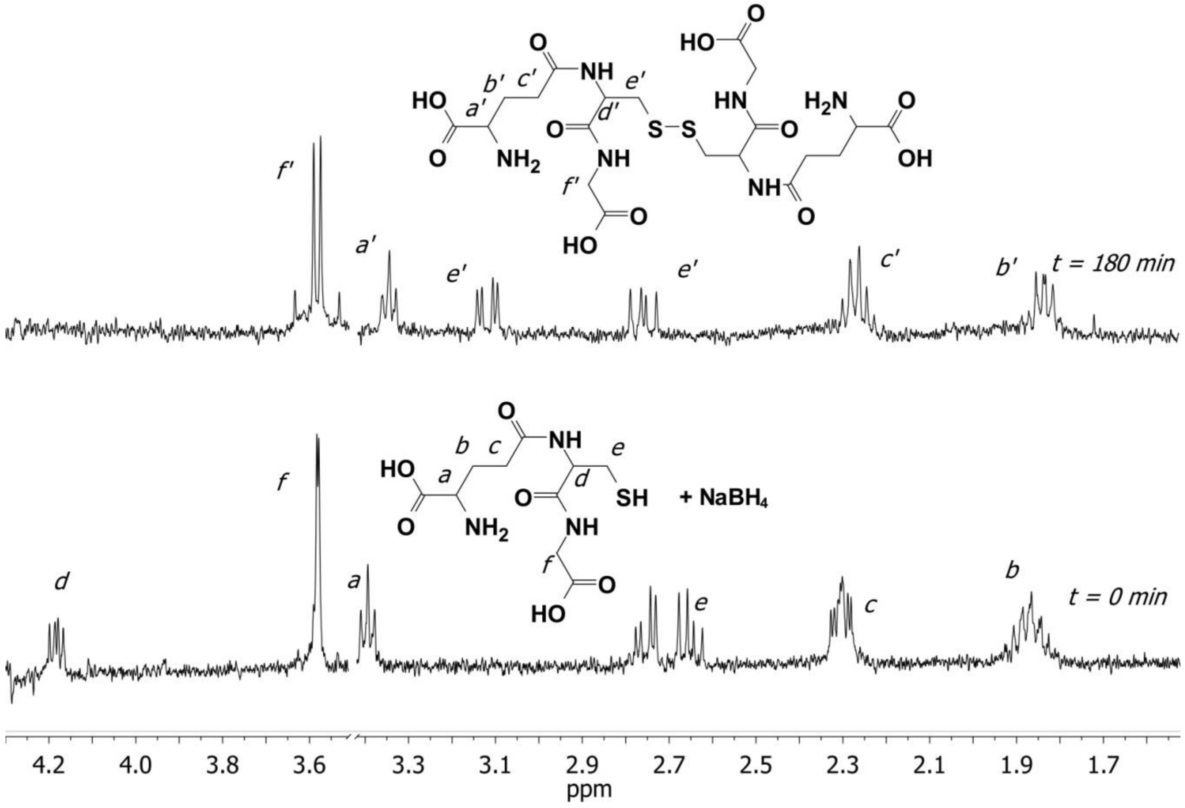

Figure S5: ^{1}H NMR spectra of the reaction mixture aliquots (GSH : $NaBH_4$ = 1 : 10, in ultrapure water/D_2O added) taken at several time points.

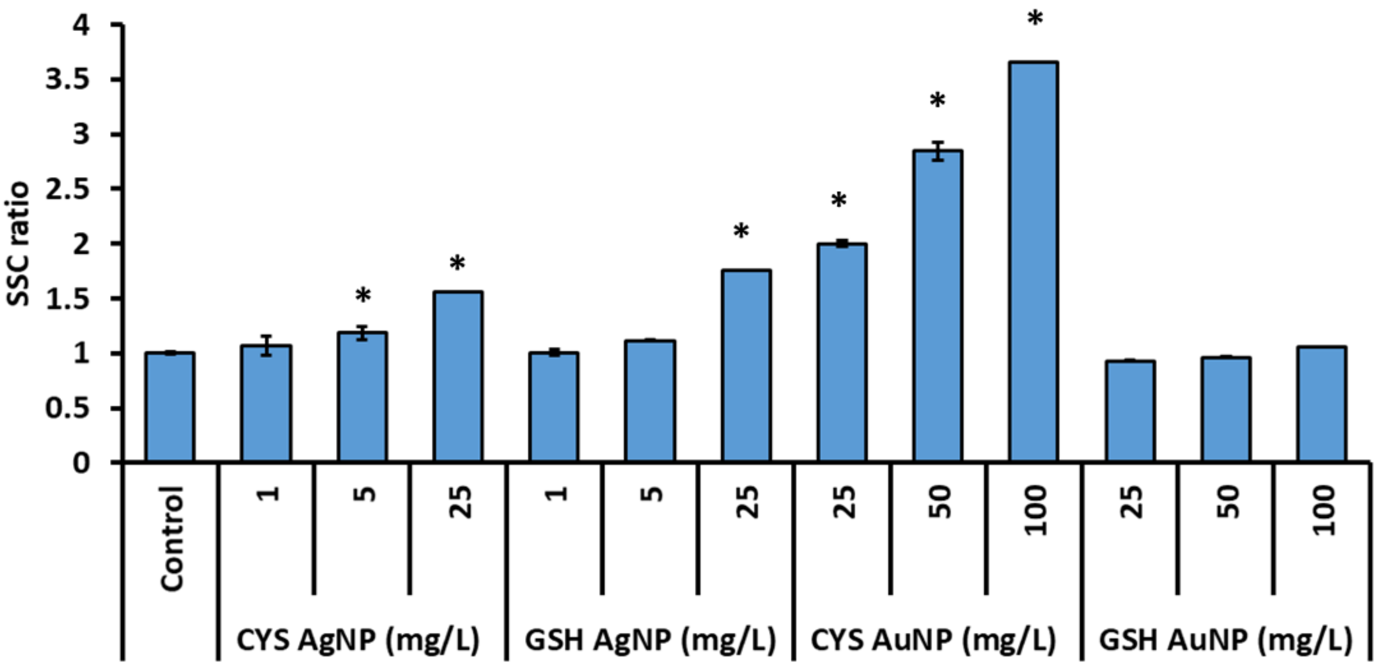

Figure S6: Uptake of silver (AgNPs) and gold nanoparticles (AuNPs) stabilized with cysteine (CYS) or glutathione (GSH) in L929 cells. Values marked with asterisk (*) differ significantly from the negative control ($P < 0.05$).

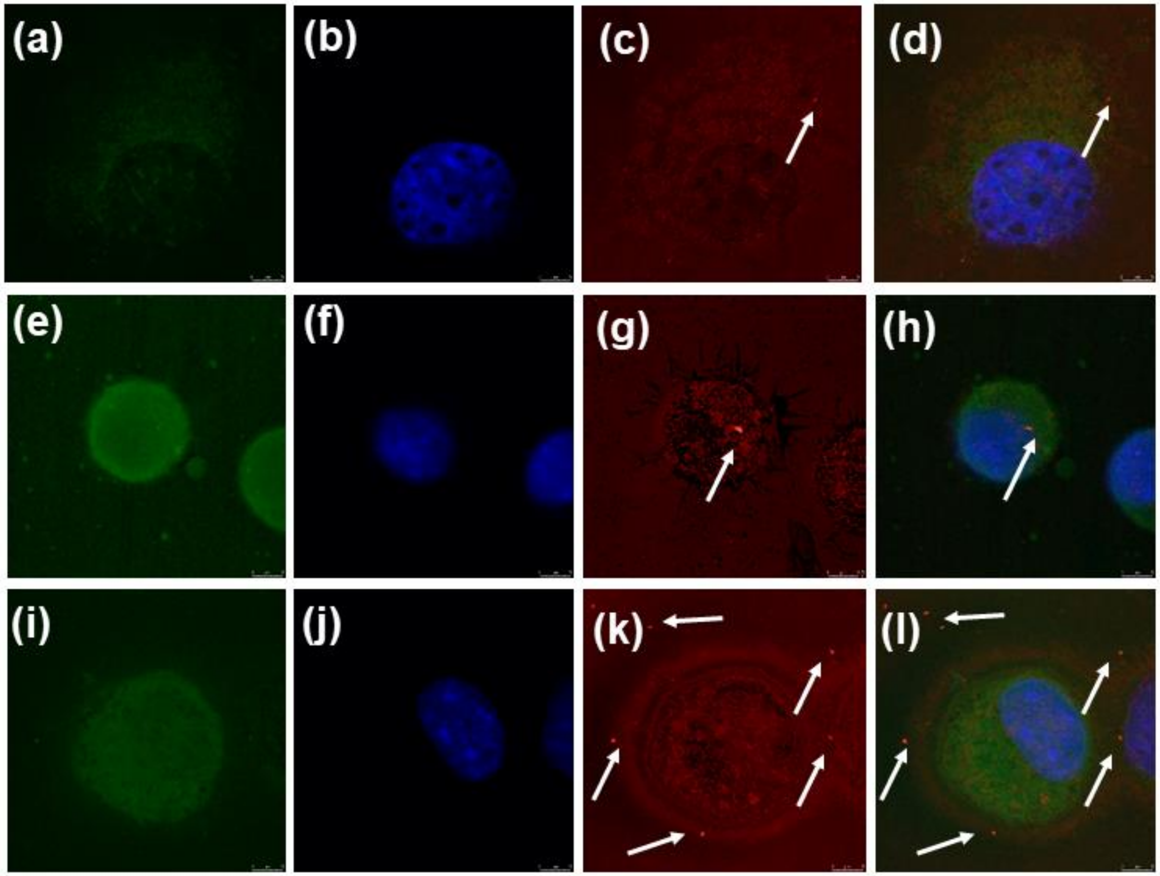

Figure S7: Visualization of cellular internalization of silver (AgNPs) and gold nanoparticles (AuNPs) stabilized with cysteine (CYS) or glutathione (GSH) in L929 cells by reflection contrast mode of confocal laser scanning microscopy. Images show maximum intensity Z-projections of cells stained with F-actin-phalloidin to visualize cell cytoskeleton (green), nucleic acid staining using Hoechst 33258 fluorescent dye (blue) and CLSM reflectance signals (red). Overlay of fluorescence stains and segmented reflectance signals are given in (d), (h) and (l) panels. Panels (a-d): Cells treated with CYS-AgNPs (at the 5 mg Ag L^{-1}). Panels (e-h): Cells treated with GSH-AgNPs (at the 5 mg Ag L^{-1}). Panels (i-l): Cells treated with GSH-AuNPs (at the 25 mg Ag L^{-1}). The NPs reflectance signals are visible as bright red signals and indicated by white arrows in panels (c-d), (g-h) and (k-l). The GSH-AuNPs can be found in the vicinity of L929 cells, but not intracellularly (k-l).

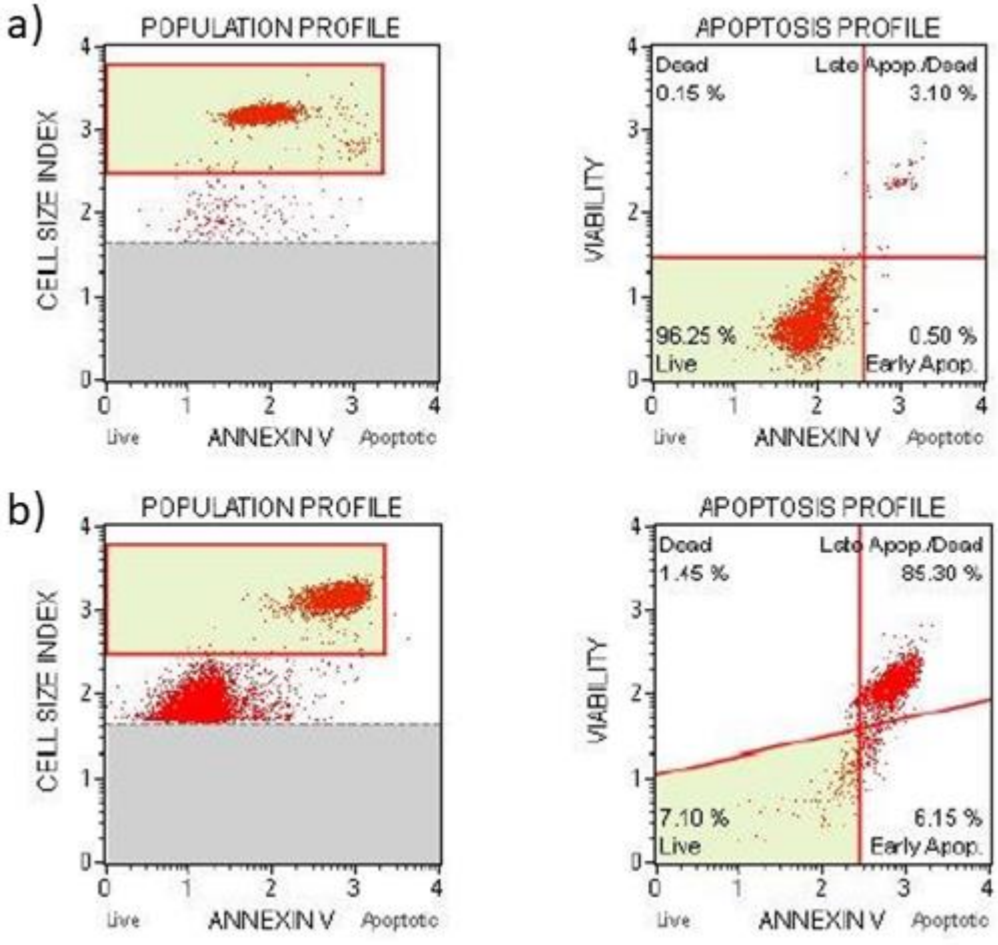

Figure S8: Population and apoptosis profiles of L929 cells analysed by flow cytometry using Muse® Annexin V and Dead Cell Assay Kit. (a) Negative controls - untreated control cells. (b) Positive controls - cells treated with paraformaldehyde (0.04 mg L^{-1}).

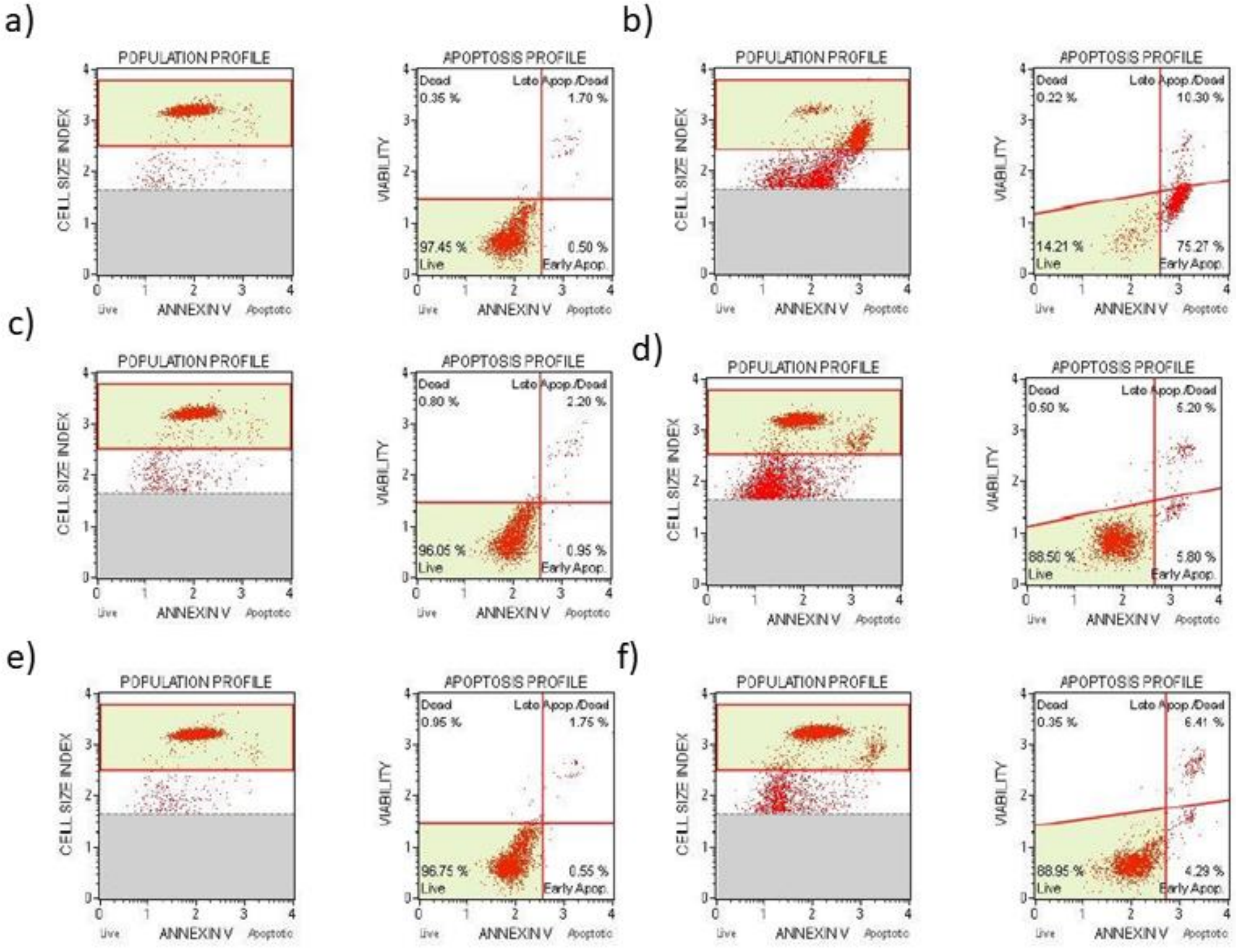

Figure S9: Population and apoptosis profiles of L9292 cells analysed by flow cytometry using Muse® Annexin V and Dead Cell Assay Kit. Cells treated with (a) 0.1 mg/L Ag^+, (b) 1 mg/L Ag^+, (c) 1 mg/L CYS-AgNPs, (d) 10 mg/L CYS-AgNPs, (e) 1 mg/L GSH-AgNPs and (f) 10 mg/L GSH-AgNPs.

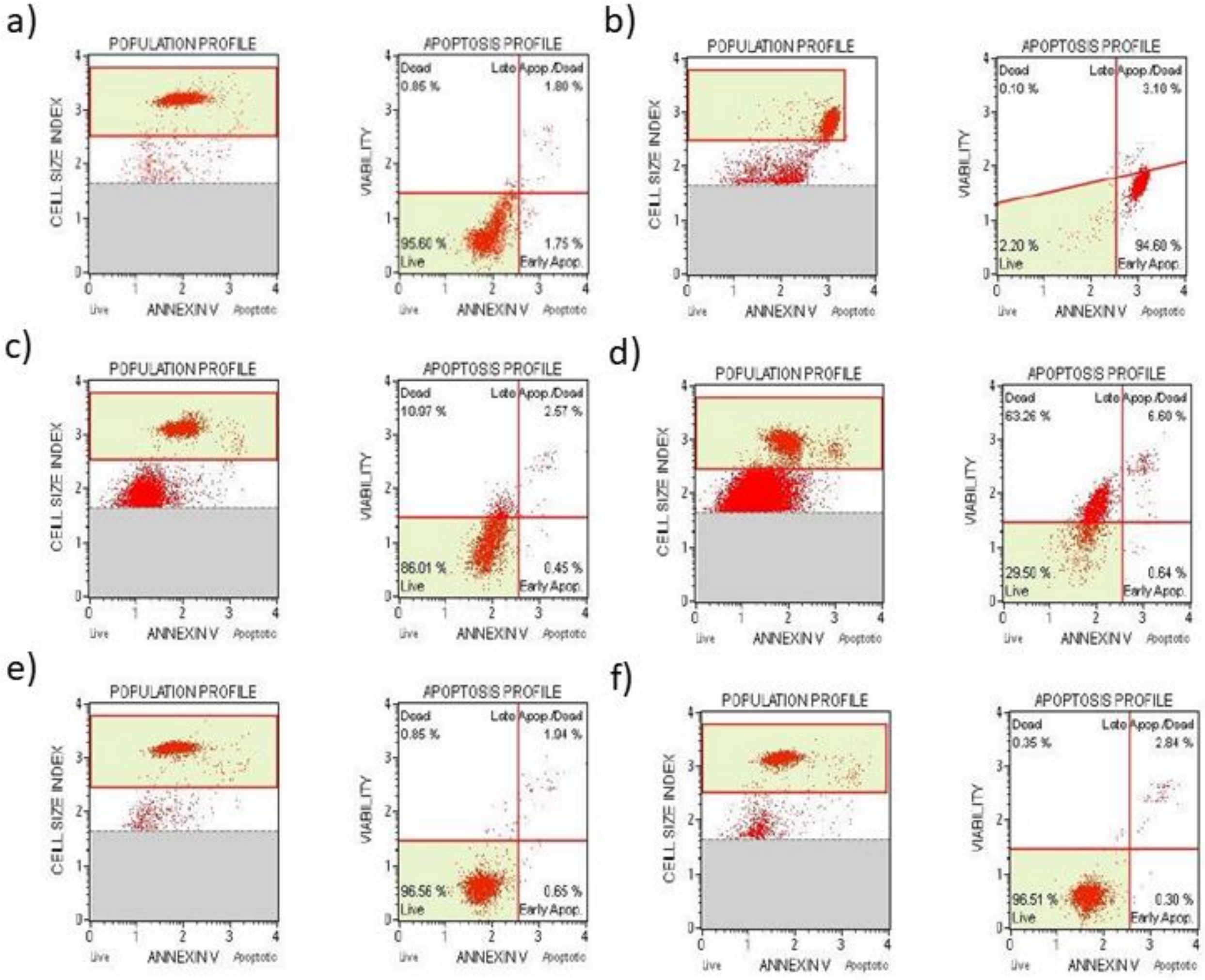

Figure S10: Population and apoptosis profiles of L9292 cells analysed by flow using Muse® Annexin V and Dead Cell Assay Kit. Cells treated with (a) 1 mg/L Au^{3+}, (b) 25 mg/L Au^{3+}, (c) 50 mg/L CYS-AuNPs, (d) 300 mg/L CYS-AuNPs, (e) 50 mg/L GSH-AuNPs and (f) 300 mg/L GSH-AuNPs.

Table S1: List of different methods employed for preparation of silver nanoparticles (AgNPs) in the presence of cysteine (CYS) and glutathione (GSH).

Biothiol	$[AgNO_3]$, mM	$[AgNO_3]/[NaBH_4]/[biothiol]$	Last added reactant	Visual observation	Size [nm] (% Volume)
CYS	1	1 : 2 : 0.2	CYS	Brown, stable	3.0 ± 0.3 (100%)
CYS	1	1 : 2 : 0.2	$NaBH_4$	Dark brown, stable	15.5 ± 2.0 (100%)
CYS	1	1 : 2 : 0.5	CYS	Brown-gray, stable	n.a.
CYS	1	1 : 2 : 0.5	$NaBH_4$	Dark gray, black precipitate	n.a.
CYS	1	1 : 10 : 0.2	CYS	Brown, unstable	3.3 ± 0.6 (100%)
CYS	1	1 : 10 : 0.2	$NaBH_4$	Brown, unstable	1.7 ± 0.8 (98%)<br>15.2 ± 1.7 (2%)
CYS	5.6	1 : 10 : 1	$NaBH_4$	Dark greenish-gray, stable but precipitates	8.0 ± 0.9 (99%)<br>30.9 ± 9.8 (<1%)
GSH	1	1 : 2 : 0.2	GSH	Brown, stable	4.8 ± 1.2 (99%)<br>51.6 ± 3.4 (<1%)
GSH	1	1 : 2 : 0.2	$NaBH_4$	Dark brown, stable	n.a.
GSH	1	1 : 2 : 0.5	GSH	Brownish-gray, stable	n.a.
GSH	1	1 : 2 : 0.5	$NaBH_4$	Dark gray, black precipitate	n.a.
GSH	1	1 : 10 : 0.2	GSH	Brownish, stable	14.6 ± 3.1 (18%)<br>46.9 ± 6.3 (82%)
GSH	5.6	1 : 10 : 1	$NaBH_4$	Brown, stable	6.0 ± 1.2 (99%)<br>315.1 ± 74.3 (<1%)

Table S2: List of different methods employed for preparation of gold nanoparticles (AuNPs) in the presence of cysteine (CYS) and glutathione (GSH).

Biothiol	$[HAuCl_4]$, mM	$[HAuCl_4]/[NaBH_4]/[biothiol]$	Last added reactant	Visual observation	Size [nm] (% Volume)
CYS	1	1 : 2 : 0.2	CYS	Dark red, turns blue-gray, unstable	n.a.
CYS	1	1 : 2 : 0.2	$NaBH_4$	Unstable	n.a.
CYS	1	1 : 2 : 1	CYS	Purple, precipitates	n.a.
CYS	1	1 : 2 : 1	$NaBH_4$	Unstable, black precipitate	n.a.
CYS	1	1 : 10 : 0.2	$NaBH_4$	Yellow, turns purple, stable	10.7 ± 3.7 (30%)<br>48.5 ± 5.8 (70%)
CYS	3	1 : 10 : 1	$NaBH_4$	Yellow, turns purple, stable but precipitates	24.2 ± 3.4 (54%)<br>80.0 ± 2.1 (10%)<br>21.7 ± 4.8 (35%)
GSH	1	1 : 2 : 0.2	GSH	Dark grey, unstable	n.a.
GSH	1	1 : 2 : 0.2	$NaBH_4$	Dark grey. unstable	n.a.
GSH	1	1 : 2 : 1	GSH	Red, unstable	n.a.
GSH	1	1 : 2 : 1	$NaBH_4$	Brown, unstable	n.a.
GSH	1	1 : 10 : 0.2	$NaBH_4$	Yellow, turns purple, stable	10.2 ± 1 (48%)<br>37.4 ± 6.5 (29%)<br>168.4 ± 67.3 (22%)
GSH	3	1 : 10 : 1	$NaBH_4$	Yellow, turns reddish, stable	6.4 ± 0.8 (65%)<br>65.1 ± 3.8 (33%)

Table S3: Overview of published data on the toxic effects of silver (AgNPs) and gold nanoparticles (AuNPs) to L929 cells *in vitro*, depending on their physico-chemical properties and concentration.

NP type	Size [nm]	ζ [mV]	Coating	Concentration applied, mg L^{-1}	Endpoint measured	Method	Result	Refe-rence
AuNP	36.3 ± 12.4	-49.4 ± 3.0	PVP	1 – 400	viability	WST-8	No reduction in viability at highest concentration	[1]
AuNP	0.8, 1.2, 1.4, 1.8, 15.0	/	TPPMS[I]	/	viability	MTT	Viability reduction in NPs of 1.8 nm or smaller; EC_{50} 250 µM, 140 µM, 56 µM, 230 µM, respectively	[2]
AuNP	19 ± 3	-35.7	citrate	1 – 25	viability	MTT	Significant cell death at 5 µg/mL or higher, after 48 h exposure, EC_{50} 18. mg L^{-1}	[3]
AgNP	/	/	PVP	25 – 100	apoptosis	annexin V/PI	>90% live cells after 24h, 5.5% early apoptotic at highest conc., no necrosis	[5]
AgNP	5	/	PVP	0.1 – 100	viability	MTT	Significant cell death at 25 µg/mL (cca 50%)	[6]

[I] triphenylphosphine monosulfonated

Table S4: Overview of the results of 48 h acute toxicity testing of gold and silver nanoparticles to *Daphnia magna* Straus, depending on their physico-chemical properties and concentration.

NP type	Size [nm]	ζ [mV]	Coating	Concentration(s) applied	Result	Reference
AgNP	55.4 ± 10.1 and 89.3 ± 21.5	-33.6±8.3 and -25.7 ± 7.9	PVP	N/A	100% mortality at 100 µg L^{-1}, no mortality below 10 µg L^{-1}	[7]
AgNP	39 ± 10	-0.13	PVP	N/A	EC_{50} 14.81 µg L^{-1}	[8]
AgNP	8.4 ± 2.8	/	PVP	N/A	EC_{50} 54.0 ± 1.4 µg L^{-1}	[9]
AgNP	35	/	PVP	N/A	EC_{50} 10.48 ± 3.23 µg L^{-1}	[10]
AgNP	40-50	-19.6 ± 3.5	carbonate	20, 50, 100, 200 and 500 mg L^{-1}	No mortality up to 500 mg L^{-1}	[11]
AgNP	28.3 ± 0.8	/	PVP	0−51 nmol L^{-1}	EC_{50} 55 ± 16 nmol L^{-1}	[12]
AuNP	21	/	bare	N/A	EC_{50} ~70 mg L^{-1}	[13]
AuNP	14 ± 4	−14 ± 8	citrate	0.1 – 10 mg L^{-1}	$EC_{10} \geq 10$ mg L^{-1}	[14]
	17.9 ± 0.9	17.9 ± 0.9	PAH[I]		40% mortality at 10 µg/L	
AuNP	12.8 ± 1.2	−15.3 ± 1.5	citrate	0.001–25 mg L^{-1}	No mortality at highest conc.	[15]
	8.0 ± 1.2	−18.5 ± 1.3	MPA[II]		No mortality at highest conc.	

[I] polyallylamine hydrochloride

[II] 3-mercaptopropionic acid

References

1. Hashimoto, M.; Yamaguchi, S.; Sasaki, J. I.; Kawai, K.; Kawakami, H.; Iwasaki, Y.; Imazato, S. *Eur. J. Oral Sci.* **2016,** *124*, 68. doi:10.1021/acsnano.6b06040
2. Pan, Y.; Neuss, S.; Leifert, A.; Fischler, M.; Wen, F.; Simon, U.; Schmid, G.; Brandau, W.; Jahnen-Dechent, W. *Small* **2007,** *3*, 1941. doi:10.1055/s-0042-112810
3. Tan, G.; Onur, M. A. *J. Biomed. Mater. Res., Part A* **2018,** *106*, 1708. doi:10.2147/IJN.S153167
4. Park, E. J.; Yi, J.; Kim, Y.; Choi, K.; Park, K. *Toxicol. In Vitro* **2010,** *24*, 872. doi:10.1007/s00204-014-1245-3
5. Wei, L.; Tang, J.; Zhang, Z.; Chen, Y.; Zhou, G.; Xi, T. *Biomed. Mater*. **2010,** *5*, 044103. doi:10.3390/ijms17091534
6. Takamiya, A. S.; Monteiro, D. R.; Bernabé, D. G.; Gorup, L. F.; Camargo, E. R.; Gomes-Filho, J. E.; Oliveira, S. H. P.; Barbosa, D. B. *J. Endod.* **2016,** *42*, 953. doi:10.1002/wnan.1322
7. Li, L. Z.; Wu, H.; Ji, C.; van Gestel, C. A. M.; Allen, H. E.; Peijnenburg, W. J. G. M. *Ecotoxicol. Environ. Saf.* **2015,** *119*, 66. doi:10.1016/j.jfda.2014.01.010
8. Newton, K. M.; Puppala, H. L.; Kitchens, C. L.; Colvin, V. L.; Klaine, S. J. *Environ. Toxicol. Chem.* **2013,** *32*, 2356. doi:10.1002/jat.2792
9. Blinova, I.; Niskanen, J.; Kajankari, P.; Kanarbik, L.; Käkinen, A.; Tenhu, H.; Penttinen, O. P.; Kahru, A. *Environ. Sci. Pollut. Res.* **2013,** *20*, 3456. doi:10.1002/jps.24001
10. Poynton, H. C.; Lazorchak, J. M.; Impellitteri, C. A.; Blalock, B. J.; Rogers, K.; Allen, H. J.; Loguinov, A.; Heckman, J. L.; Govindasmawy, S. *Environ. Sci. Technol.* **2012,** *46*, 6288. doi:10.1897/08-002.1
11. Zhao, C.-M.; Wang, W.-X. *Environ. Toxicol. Chem.* **2011,** *30*, 885. doi:10.4491/eer.2010.15.1.428
12. Khan, F. R.; Paul, K. B.; Dybowska, A. D.; Valsami-Jones, E.; Lead, J. R.; Stone, V.; Fernandes, T. F. *Environ. Sci. Technol.* **2015,** *49*, 4389. doi:10.1002/etc.703
13. Li, T.; Albee, B.; Alemayehu, M.; Diaz, R.; Ingham, L.; Kamal, S.; Rodriguez, M.; Whaley Bishnoi, S. *Anal. Bioanal. Chem.* **2010,** *398*, 689. doi:10.1016/j.tiv.2011.03.008
14. Skjolding, L. M.; Kern, K.; Hjorth, R.; Hartmann, N.; Overgaard, S.; Ma, G.; Veinot, J. G. C.; Baun, A. *Ecotoxicology* **2014,** *23*, 1172. doi:10.1021/es062629t
15. Bozich, J. S.; Lohse, S. E.; Torelli, M. D.; Murphy, C. J.; Hamers, R. J.; Klaper, R. D. *Environ. Sci.: Nano* **2014,** *1*, 260. doi:10.1021/es8026314
